# Promotion of mitochondrial biogenesis by necdin protects neurons against mitochondrial insults

**DOI:** 10.1038/ncomms10943

**Published:** 2016-03-14

**Authors:** Koichi Hasegawa, Toru Yasuda, Chinatsu Shiraishi, Kazushiro Fujiwara, Serge Przedborski, Hideki Mochizuki, Kazuaki Yoshikawa

**Affiliations:** 1 Laboratory of Regulation of Neuronal Development, Institute for Protein Research, Osaka University, Suita, Osaka 565-0871, Japan; 2 Department of Neurology, Graduate School of Medicine, Osaka University, Suita, Osaka 565-0871, Japan; 3 Department of Neurology, Pathology and Cell Biology, Columbia University, New York, New York, 10032, USA

## Abstract

Neurons rely heavily on mitochondria for their function and survival. Mitochondrial dysfunction contributes to the pathogenesis of neurodegenerative diseases such as Parkinson's disease. PGC-1α is a master regulator of mitochondrial biogenesis and function. Here we identify necdin as a potent PGC-1α stabilizer that promotes mitochondrial biogenesis via PGC-1α in mammalian neurons. Expression of genes encoding mitochondria-specific proteins decreases significantly in necdin-null cortical neurons, where mitochondrial function and expression of the PGC-1α protein are reduced. Necdin strongly stabilizes PGC-1α by inhibiting its ubiquitin-dependent degradation. Forced expression of necdin enhances mitochondrial function in primary cortical neurons and human SH-SY5Y neuroblastoma cells to prevent mitochondrial respiratory chain inhibitor-induced degeneration. Moreover, overexpression of necdin in the substantia nigra *in vivo* of adult mice protects dopaminergic neurons against degeneration in experimental Parkinson's disease. These data reveal that necdin promotes mitochondrial biogenesis through stabilization of endogenous PGC-1α to exert neuroprotection against mitochondrial insults.

## Abstract (web summary)

Mitochondrial dysfunction occurs in Parkinson's disease, although the underlying mechanisms are unclear. Here, the authors find necdin works to stabilise the mitochondrial regulator PGC-1α, and that overexpression of necdin protects against MPTP-mediated neurodegeneration both *in vitro* and *in vivo*.

## Graphical abstract

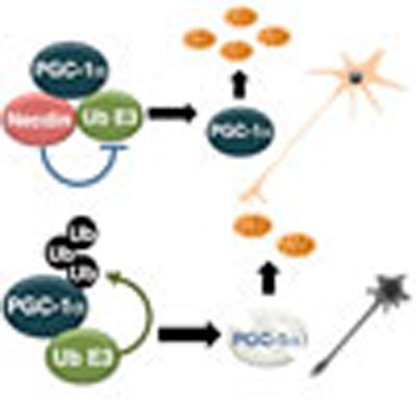

Mammalian neurons require high mitochondrial activities to generate a large amount of ATP for their signalling events such as action potential generation and excitatory synaptic transmission^1^. Mitochondria are also involved in neuronal death and contribute to neuroprotection against various detrimental stresses^2^. Furthermore, mitochondrial abnormalities are suggested to contribute to the pathogenesis of neurodegenerative diseases such as Parkinson's disease (PD), amyotrophic lateral sclerosis (ALS), Alzheimer's disease and Huntington's disease^3^,^4^. However, little is known about the regulatory mechanisms of mitochondrial biogenesis in mammalian neurons under physiological and pathological conditions.

The peroxisome proliferator-activated receptor γ coactivator-1 (PGC-1) family, which consists of PGC-1α, PGC-1β and PRC, plays a central role in governing a transcriptional regulatory network for mitochondrial biogenesis and respiratory function^5^. The PGC-1 family transcriptional coactivators enhance the activities of the nuclear respiratory factors NRF1 and NRF2, which induce transactivation of many genes encoding mitochondria-specific proteins involved in respiratory chain, mitochondrial DNA transcription/replication and protein import/assembly^6^. PGC-1α is the first identified PGC-1 family member^7^, and its expression and function have been most extensively studied^5^. In non-neuronal cells, expression of PGC-1α is dynamically regulated at the transcriptional and post-translational levels in response to various environmental stimuli such as temperature, nutritional status and physical activity^5^,^8^. However, there is limited information on the regulation of neuronal PGC-1α and its involvement in mitochondrial biogenesis.

Necdin is a MAGE (melanoma antigen) family protein originally isolated from neurally differentiated embryonal carcinoma cells^9^. Necdin is expressed in virtually all neurons throughout the nervous system^10^. The *necdin* gene (gene symbols; *Ndn* for mouse, *NDN* for human) is expressed only from the paternal allele via genomic imprinting, a mammal-specific epigenetic regulation of gene expression^11^,^12^. Necdin interacts with the major transcription factors E2F1 and p53 to suppress cell proliferation and apoptosis^13^,^14^,^15^,^16^. Moreover, necdin binds to Sirt1, an NAD^+^-dependent protein deacetylase involved in the regulation of energy homeostasis, and facilitates Sirt1-mediated deacetylation of the transcription factors p53 and FoxO1 in neurons^16^,^17^. These findings suggest that necdin interacts with major nuclear proteins to modulate the transcriptional regulation networks in mammalian neurons.

We here report that necdin facilitates neuronal mitochondrial biogenesis via PGC-1α stabilization by suppressing its proteolytic degradation in the ubiquitin-proteasomal system. Necdin forms a stable complex with PGC-1α in the nucleus of cortical neurons to maintain high mitochondrial activities. Furthermore, we demonstrate that necdin exerts potent neuroprotective effects on dopaminergic neurons against mitochondrial complex I inhibitors that are commonly used for modelling PD^18^. Our findings will provide a better understanding of the regulatory mechanism underlying neuronal mitochondrial biogenesis under physiological and pathological conditions.

## Results

### Necdin promotes neuronal mitochondria-related gene expression

**Figure 1:**
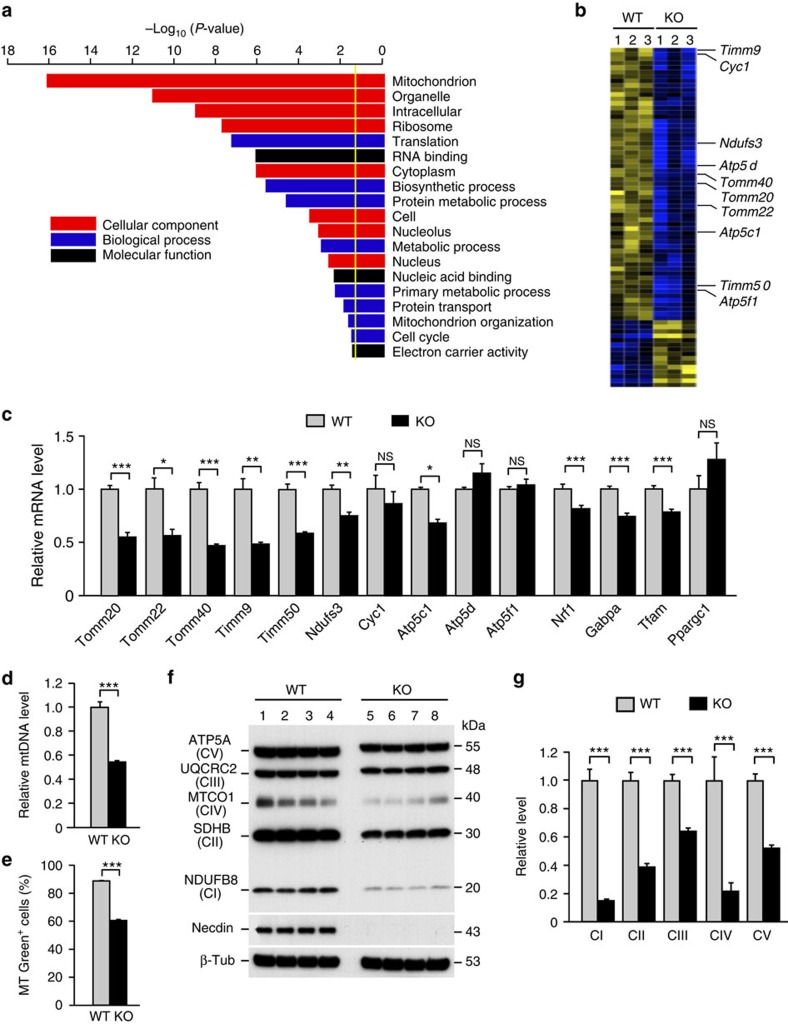
Necdin promotes the expression of mitochondria-related genes in primary cortical neurons. (**a**) Gene expression profiling of primary cortical neurons prepared from wild-type and necdin-null mice at E14.5 was performed using DNA microarrays. Enriched gene ontology terms of genes whose expression decreased (>20% at *P*<0.05) in necdin-null cortical neurons. *P* value cutoff (yellow line), 1.3=−log_10_(0.05). (**b**) Clustered mitochondrion-related genes whose expression changed significantly (*P*<0.05) between wild-type (WT) and necdin-null (knockout; KO) neurons (yellow, up; blue, down; *n*=3). Full data are in Supplementary Table 1. (**c**) Expression levels of mitochondria-related genes and mitochondrial biogenesis-regulatory genes in cortical neurons were determined by qRT–PCR (*n*=3–6). (**d**) *D-loop* DNA (mtDNA) levels were measured by qRT–PCR and normalized to nuclear gene *Rplp0* levels (*n*=4). (**e**) MitoTracker Green FM (MT Green) fluorescence levels were quantified by fluorescence-activated cell sorting analysis (*n*=4). Mean fluorescence intensity: 367 (WT); 268 (KO). (**f**,**g**) Expression of oxidative phosphorylation complexes I–V subunits was analysed by immunoblotting using antibodies against the enzyme subunits indicated (**f**), and these bands were quantified by densitometry (*n*=4) (**g**). Data represent means±s.e.m.; *P<0.05, **P<0.01, ***P<0.005, NS, not significant at *P*≥0.05; Student's *t*-test.

To investigate whether necdin modulates specific gene transcription networks in brain neurons, we performed microarray-based gene expression profiling in necdin-null cortical neurons (GEO accession; GSE63498). In gene ontology analysis for reduced gene expression in necdin-null neurons, the term mitochondrion in the cellular component category was the most significantly enriched (Fig. 1a). Of 61 downregulated genes (Fig. 1b and Supplementary Table 1), 10 genes encoding mitochondria-specific proteins were selected, and their expression levels were determined by quantitative reverse transcription–PCR (qRT–PCR) (Fig. 1c). In necdin-null neurons, the mRNA levels of *Tomm20*, *Tomm22*, *Tomm40*, *Timm9* and *Timm50*, which encode mitochondrial import receptors, decreased by 41–53%, and those of *Ndufs3* and *Atp5c1*, which encode electron transport chain-associated enzyme components, decreased by 24% and 32%, respectively. However, *cytochrome c* (*Cyc1*), *Atp5d* and *Atp5f1* mRNA levels were unchanged. We also quantified the expression of mitochondrial biogenesis-regulatory genes by qRT–PCR. *Nrf1*, *Gabpa* (also known as *Nrf2*) and *Tfam* mRNA levels decreased significantly in necdin-null neurons, whereas no significant change in *PGC-1α* (*Ppargc1*) mRNA expression was observed. The gene expression signature of necdin-null neurons indicates that necdin promotes mitochondrial biogenesis in cortical neurons.

### Necdin promotes neuronal mitochondrial biogenesis

We then examined whether mitochondrial amounts and activities are reduced in necdin-null neurons. The levels of mitochondrial DNA (*D-loop*) and the mitochondrial marker MitoTracker Green FM, which localizes to mitochondria regardless of mitochondrial membrane potential, were reduced by 46% and 31%, respectively, in necdin-null neurons (Fig. 1d,e). The levels of oxidative phosphorylation complexes I–V (CI–CV) subunits were also reduced in necdin-null neurons by 85% (CI, NDUFB8), 61% (CII, SDHB), 36% (CIII, UQCRC2), 78% (CIV, MTCO1) and 47% (CV, ATP5A) (Fig. 1f,g). These results suggest that endogenous necdin promotes mitochondrial biogenesis in cortical neurons.

**Figure 2:**
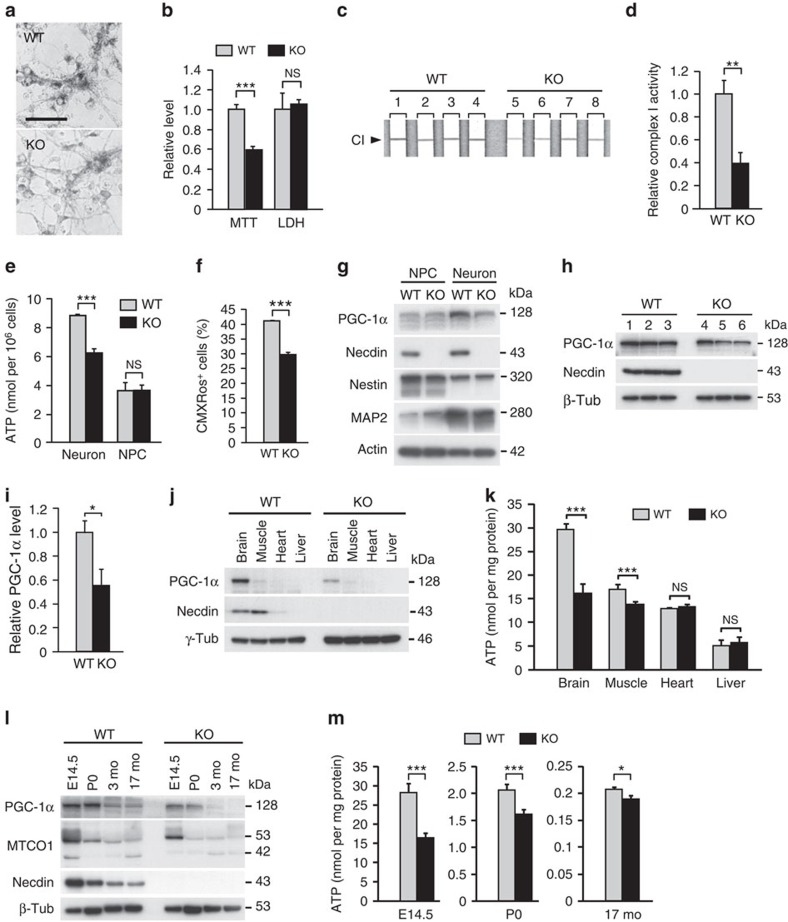
Necdin deficiency reduces neuronal mitochondrial function and PGC-1α protein expression. (**a**) MTT-formazan deposits in primary cortical neurons from wild-type (WT) and necdin-null (knockout; KO) mice were observed by differential interference contrast microscopy after incubation with MTT for 90 min. Scale bar, 50 μm. (**b**) MTT-formazan levels in primary neurons and released LDH levels were measured after incubation with MTT for 4 h (WT, *n*=5; KO, *n*=7). (**c**,**d**) Complex I (CI, arrowhead) in neuronal extracts of WT and KO mice were immunocaptured for NADH oxidizing activity (**c**). The product signals were quantified by densitometry (**d**) (*n*=4). (**e**) ATP levels in neurons and NPCs were measured by chemiluminescence assay (*n*=4). (**f**) CMXRos fluorescence levels were quantified by fluorescence-activated cell sorting analysis (*n*=3). Mean fluorescence intensity: 116 (WT); 96 (KO). (**g**) Expression of PGC-1α, necdin, nestin, MAP2 and actin in NPCs and neurons prepared from E14.5 mouse cortex was analysed by western blotting. (**h**,**i**) PGC-1α expression in cortical neurons was analysed by immunoblotting (**h**) and quantified by densitometry (**i**) (*n*=3). (**j**) PGC-1α and necdin expression levels in the brain, gastrocnemius muscle, heart and liver prepared from WT and KO mice at E14.5 were analysed by western blotting. (**k**) ATP levels in the tissues indicated were measured by chemiluminescence assay (*n*=4). (**l**) Expression of PGC-1α, MTCO1 and necdin in the brain of WT and KO mice at different ages was analysed by western blotting. (**m**) ATP levels in the brain at indicated ages were measured by chemiluminescence assay (*n*=5). mo, months old. Data represent means±s.e.m.; *P<0.05, **P<0.01, ***P<0.005, NS, not significant at *P*≥0.05; Student's *t*-test.

We determined the effect of necdin on mitochondrial metabolic activity in primary cortical neurons using 3-[4,5-dimethylthiazol-2-yl]-2,5 diphenyl tetrazolium bromide (MTT), a tetrazolium dye metabolized into water-insoluble formazan mainly in mitochondria. Necdin-null primary neurons exhibited normal morphology, but contained reduced MTT-formazan deposits (Fig. 2a). In quantitative MTT assay, MTT-formazan levels in necdin-null neurons decreased by 41% in the conditions where cellular damage was absent as assessed by lactate dehydrogenase (LDH) release (Fig. 2b). We further analysed oxidative phosphorylation complex I activity in primary cortical neurons by immunocapture assay. Complex I activity was reduced in necdin-null neurons by 60% (Fig. 2c,d). Cellular ATP levels were also significantly reduced in necdin-null neurons, but not in necdin-null neural progenitor cells (NPCs), indicating that necdin promotes the mitochondrial activity predominantly in differentiated neurons (Fig. 2e). We then examined whether mitochondrial membrane potential is reduced in necdin-null neurons. The levels of chloromethyl-X-rosamine, a mitochondrial membrane potential-dependent fluorescent probe, were reduced by 28% in necdin-null neurons (Fig. 2f).

PGC-1α serves as a master regulator of mitochondrial biogenesis in mammalian cells^5^,^7^. Because we found no significant change in *PGC-1α* expression at the mRNA level in necdin-null neurons, we analysed the expression of PGC-1α at the protein level using a novel antibody raised against mouse PGC-1α (PGCAN) (Supplementary Fig. 1). In NPCs and cortical neurons, PGC-1α was detected as a major 128-kDa band by western blotting, and wild-type cortical neurons expressed higher PGC-1α levels than wild-type NPCs and necdin-null cortical neurons (Fig. 2g). Furthermore, the expression level of PGC-1α in necdin-null neurons was 55% of the wild-type control level (Fig. 2h,i). We then analysed the expression of the PGC-1α protein in various tissues at embryonic day 14.5 (E14.5) by western blotting (Fig. 2j). PGC-1α was clearly detected in the brain, slightly in the skeletal muscle (gastrocnemius) and hardly detectable in the heart and liver. ATP levels in the brain and muscle, where necdin was highly expressed, were significantly lower in necdin-null mice than in wild-type mice at E14.5 (Fig. 2k). The brain levels of PGC-1α were the highest at E14.5 and postnatal day 0 (P0), and reduced markedly at adult stages (Fig. 2l). Expression levels of MTCO1 and necdin were the highest at E14.5 and decreased during postnatal development. Necdin-null mice had lower levels of PGC-1α and MTCO1 than wild-type mice throughout brain development. Interestingly, ATP levels in the brain also decreased sharply during development (<10% and <1% of the E14.5 level at P0 and 17 months, respectively) and were significantly reduced in the brain of necdin-deficient mice (Fig. 2m). We further examined the effect of necdin on mitochondrial degradation in cortical neurons using carbonyl cyanide *m*-chlorophenyl hydrazone, a mitochondrial uncoupler, as described previously^19^. There was no difference in the expression levels of the autophagy marker LC3-II between wild-type and necdin-null neurons treated with carbonyl cyanide *m*-chlorophenyl hydrazone (Supplementary Fig. 2), suggesting that necdin fails to affect mitochondrial degradation.

### Necdin and PGC-1α are colocalized in neuronal nucleus

**Figure 3:**
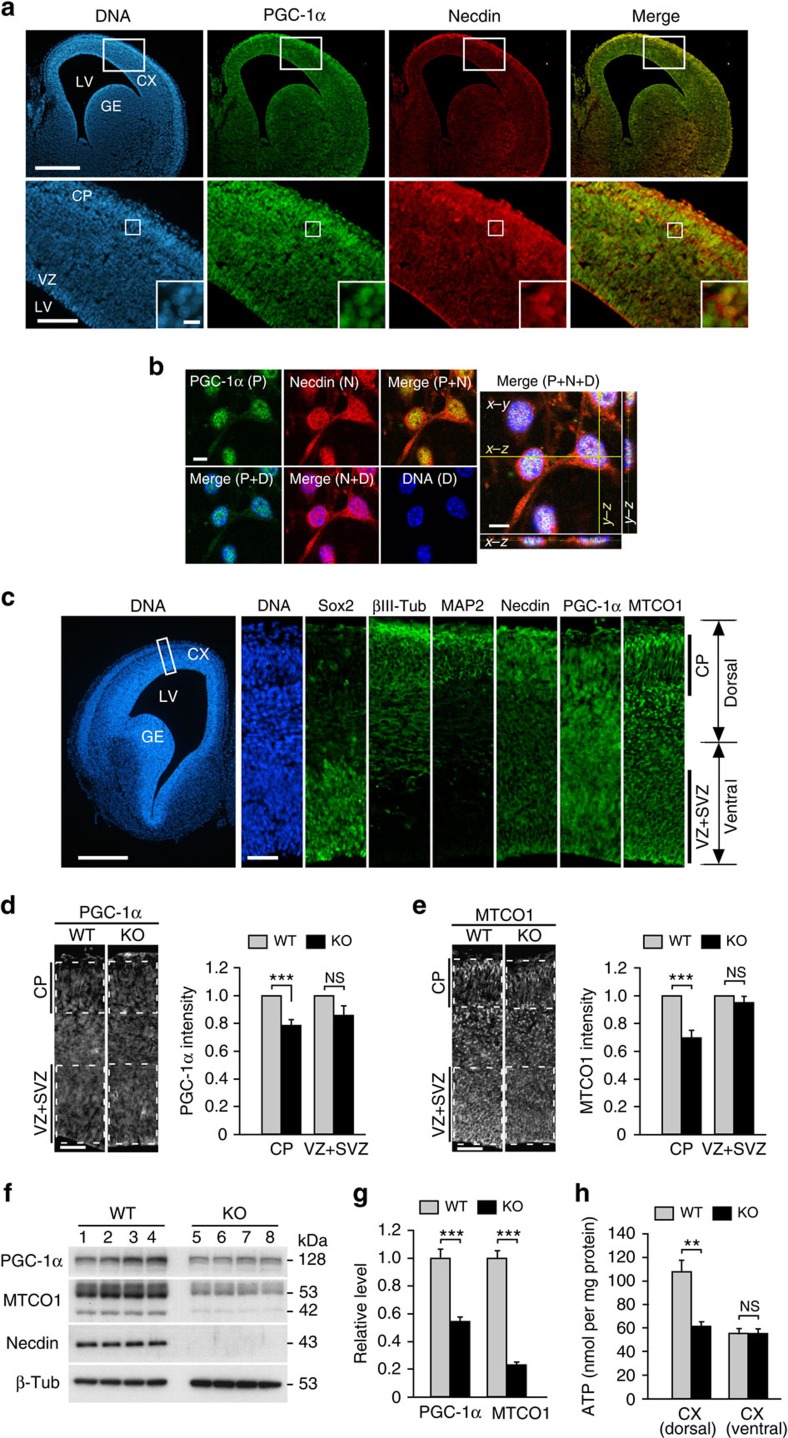
Necdin and PGC-1α are colocalized in the nucleus of cortical neurons. (**a**) Forebrain sections were prepared from E14.5 mice and double-immunostained for PGC-1α and necdin. Nuclear DNA was counterstained with Hoechst 33342. Boxed areas in the upper and lower panels are magnified in the lower panels and insets, respectively. Scale bars, 500 μm (upper), 100 μm (lower) and 10 μm (insets). (**b**) Primary neurons were triply stained for PGC-1α, necdin and DNA, and observed by confocal laser-scanning microscopy. Confocal images with *Z*-stacks in accessory panels were merged (right). Scale bars, 10 μm. (**c**) Sox2, βIII-tubulin (βIII-Tub), MAP2, necdin, PGC-1α and MTCO1 in E14.5 mouse cortical region were analysed by fluorescence immunohistochemistry. Scale bars; 500 μm (first) and 50 μm (second). (**d**,**e**) Fluorescence intensities of PGC-1α (**d**) and MTCO1 (**e**) in the CP and VZ+SVZ areas of wild-type (WT) and necdin-null (KO) E14.5 mice were quantified by fluorescence microphotometry (PGC-1α, *n*=4; MTCO1, *n*=5; right). Scale bars, 50 μm. (**f**,**g**) PGC-1α and MTCO1 in E14.5 mouse cortex extracts were analysed by immunoblotting (**f**) and quantified by densitometry (**g**) (*n*=4). (**h**) The cortex of E14.5 mice was manually dissected into the ventral and dorsal parts, and the ATP levels were measured (*n*=3). Data represent means±s.e.m.; **P<0.01, ***P<0.005, NS, not significant at *P*≥0.05; Student's *t*-test. CP, cortical plate; CX, cortex; GE, ganglionic eminence; KO, knockout; LV, lateral ventricle; VZ, ventricular zone; VZ+SVZ, ventricular and subventricular zones.

We next investigated the distribution patterns of necdin and PGC-1α in the neocortex by immunohistochemistry. In E14.5 mouse forebrain, virtually all cortical neurons expressed both PGC-1α and necdin (Fig. 3a). Most PGC-1α-expressing cells overlapped with necdin-expressing cells in the cortical plate where differentiated neurons are present. PGC-1α and necdin were colocalized in the nucleus of primary cortical neurons as analysed by confocal laser-scanning microscopy (Fig. 3b). Furthermore, fluorescence microphotometry revealed that PGC-1α and MTCO1 immunoreactivities were significantly reduced in the neocortex of necdin-null mice (Fig. 3c–e). These reductions were observed in the cortical plate but not in the ventricular/subventricular zone, where undifferentiated neural precursors are present. Western blot analysis revealed that expression of PGC-1α and MTCO1 decreased by 46% and 77%, respectively, in necdin-null forebrain extracts (Fig. 3f,g). Tissue ATP levels were markedly reduced in the dorsal cortical area, which contains the cortical plate, of necdin-null mice but not in the ventral area containing undifferentiated cell populations (Fig. 3h). These data suggest that necdin upregulates the protein levels of PGC-1α and MTCO1 in the brain *in vivo*.

### Necdin binds and stabilizes PGC-1α

**Figure 4:**
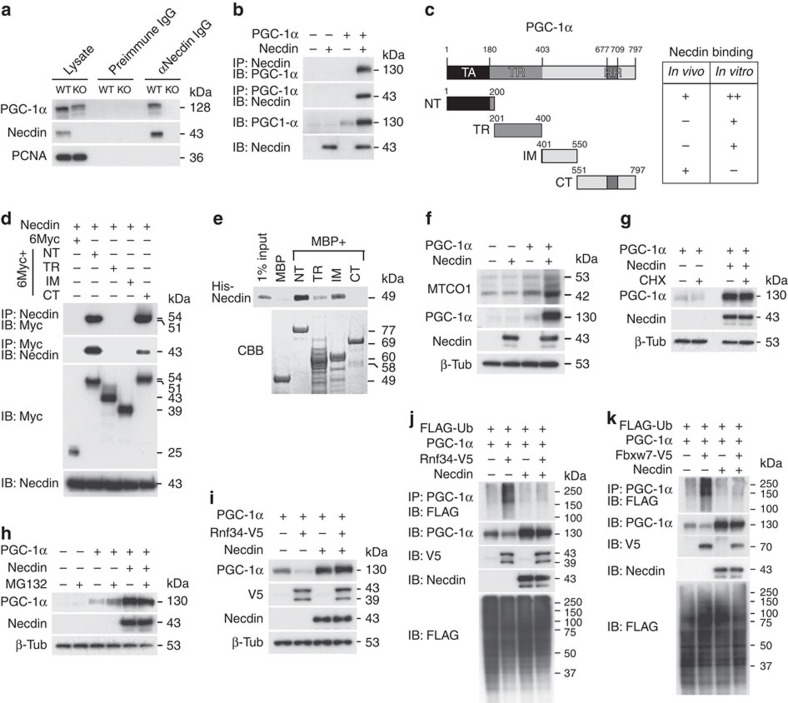
Necdin stabilizes PGC-1α by inhibiting its ubiquitin-proteasomal degradation. (**a**) Forebrain extracts from E14.5 wild-type (WT) and necdin-null (KO) mice were immunoprecipitated with anti-necdin IgG and control preimmune IgG for endogenous binding between necdin and PGC-1α. PCNA, negative control. (**b**) Necdin and PGC-1α expressed in cDNA-transfected HEK293A cells were immunoprecipitated and immunoblotted. (**c**) Diagram of the PGC-1α deletion mutants. (**d**) Necdin and 6Myc-tagged PGC-1α deletion mutants in transfected HEK293A cells were immunoprecipitated and immunoblotted. (**e**) Bacterially synthesized MBP-fused PGC-1α deletion mutants were incubated with His-tagged necdin (His-Necdin). Bound His-Necdin was detected by immunoblotting. Recombinant proteins were stained with Coomassie Brilliant Blue (CBB). Results are summarized in **c** (right). (**f**) MTCO1 levels in HEK293A cells transfected with PGC-1α and necdin cDNAs were detected 72 h post transfection. (**g**,**h**) PGC-1α levels in transfected HEK293A were analysed 24 h post transfection after treating with CHX for 60 min (CHX+) (**g**) or MG132 for 3 h (MG132+) (**h**) before harvest. (**i**) HEK293A cells were transfected with cDNAs for PGC-1α, V5-tagged Rnf34 (Rnf34-V5) and necdin, and incubated for 24 h. PGC-1α, V5, necdin and β-tubulin were detected by immunoblotting. (**j**,**k**) Effects of necdin on PGC-1α degradation and PGC-1α ubiquitination mediated by Rnf34 (**j**) or Fbxw7 (**k**) were analysed using HEK293A cells transfected with cDNAs for PGC-1α, necdin, V5-tagged Rnf34 (Rnf34-V5), V5-tagged Fbxw7 (Fbxw7-V5) and FLAG-tagged ubiquitin (FLAG-Ub). Ubiquitination of immunoprecipitated PGC-1α was detected with anti-FLAG antibody. CT, C-terminal; IB, immunoblotted; IM, intermediate; IP, immunoprecipitated; KO, knockout; NT, N-terminal; RR, RNA recognition; TA, transcriptional activation; TR, transcriptional repression.

We next examined the interaction between necdin and PGC-1α by co-immunoprecipitation assay. Endogenous PGC-1α was coprecipitated with necdin in forebrain extracts prepared from wild-type mice, but not from necdin-null mice (Fig. 4a). Co-immunoprecipitation assay using transfected HEK293A cells revealed that PGC-1α was coprecipitated with necdin, which was conversely coprecipitated with PGC-1α (Fig. 4b). Remarkably, PGC-1α expression levels in transfected HEK293A cells were markedly increased by coexpression of necdin, suggesting that the PGC-1α protein expressed in HEK293A cells is strongly stabilized in the presence of necdin. Necdin bound to the N-terminal region of PGC-1α in immunoprecipitation assay and *in vitro* pull-down assays (Fig. 4c–e). We also analysed the subcellular localization of PGC-1α in transfected HEK293A cells by immunocytochemistry (Supplementary Fig. 3). Necdin was colocalized with PGC*-*1α in the nucleus of co-transfected HEK293A cells, and coexpressed necdin significantly increased nuclear PGC-1α levels. We then examined whether forced expression of necdin and PGC-1α increases mitochondrial biogenesis in HEK293A cells (Fig. 4f). Necdin failed to increase the expression of MTCO1 but promoted PGC-1α-mediated MTCO1 expression (untransfected control, 1.0±0.2; necdin, 0.9±0.0; PGC-1α, 2.5±0.5; necdin+PGC-1α, 5.8±0.7; control versus PGC-1α, *P*<0.05; PGC-1α versus necdin+PGC-1α, *P*<0.01, by analysis of variance with Tukey–Kramer *post hoc* test, *n*=3 per each group).

We also examined whether necdin affects the stabilization of PGC-1β, another PGC family member expressed in mammalian cortical neurons^20^,^21^, using HEK293A cells expressing necdin and Myc-tagged PGC-1β or Myc-tagged PGC-1α (positive control; Supplementary Fig. 4). PGC-1β, like PGC-1α, was stabilized by necdin, which promoted PGC-1β-mediated MTCO1 expression, suggesting that necdin requires these PGC-1 family proteins for promoting mitochondrial biogenesis.

### Necdin inhibits ubiquitin-dependent degradation of PGC-1α

To clarify whether necdin inhibits proteolytic degradation of PGC-1α by the ubiquitin-proteasome pathway, we examined the effects of the protein synthesis inhibitor cycloheximide (CHX) and the proteasome inhibitor MG132 on PGC-1α expression levels in transfected HEK293A cells. Necdin markedly increased the PGC-1α level irrespective of the presence or absence of CHX (Fig. 4g) or MG132 (Fig. 4h), indicating that necdin strongly inhibits the degradation of PGC-1α in the proteasome. We then examined the effect of necdin on ubiquitin-dependent degradation of PGC-1α using Rnf34, a PGC-1α E3 ubiquitin ligase^22^. Rnf34 reduced the PGC-1α level, and necdin completely inhibited the reduction (Fig. 4i). In addition, necdin strongly suppressed Rnf34-mediated ubiquitination of PGC-1α (Fig. 4j). Necdin also protected PGC-1α against ubiquitination mediated by Fbxw7, another PGC-1α E3 ubiquitin ligase^23^ (Fig. 4k). These data indicate that necdin stabilizes PGC-1α by inhibiting its degradation in the ubiquitin-proteasomal system.

### Necdin prevents oligomycin-induced neurodegeneration

**Figure 5:**
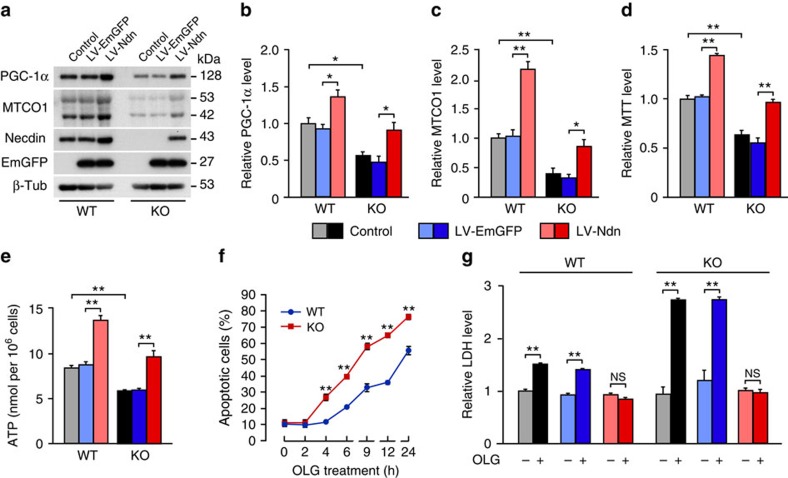
Necdin prevents oligomycin-induced neurodegeneration of primary cortical neurons. (**a**) Primary cortical neurons prepared from wild-type (WT) and necdin-null (knockout; KO) mice at E14.5 were infected with LV expressing EmGFP (LV-EmGFP) or necdin (LV-Ndn), and expression of PGC-1α, MTCO1 and necdin was analysed by western blotting. Control, uninfected neurons. (**b**,**c**) PGC-1α (**b**) and MTCO1 (**c**) levels were quantified by densitometry (*n*=4). (**d**,**e**) MTT (**d**) and ATP (**e**) levels in LV-infected cortical neurons were measured. (**f**) Oligomycin (OLG)-induced neuronal apoptosis was analysed by Hoechst 33342 staining for condensed or fragmented nuclei at the times indicated (*n*=3). Data represent means±s.e.m.; **P<0.01; Student's *t*-test. (**g**) LV-infected cortical neurons were treated without (−) or with (+) OLG for 6 h, and released LDH levels were measured. Data represent means±s.e.m. (*n*=3–4; **b**–**e**,**g**); *P<0.05, **P<0.01, NS, not significant at *P*≥0.05; analysis of variance with Tukey–Kramer *post hoc* test.

To examine whether necdin promotes mitochondrial biogenesis and function, we transferred the mouse *necdin* gene into primary cortical neurons using lentivirus vectors (LVs). LV-mediated overexpression of necdin increased the PGC-1α levels by 49% and 91% in wild-type and necdin-null neurons, respectively, as compared with the Emerald Green Fluorescent Protein (EmGFP) control levels (Fig. 5a,b). Similarly, necdin overexpression increased the MTCO1 levels 2.1- and 2.7-fold in wild-type and necdin-null neurons, respectively (Fig. 5c). We also examined the effects of necdin overexpression on mitochondrial activities in primary cortical neurons. LV-mediated overexpression of necdin increased MTT levels by 43 and 73% in wild-type and necdin-null neurons, respectively (Fig. 5d). Similarly, necdin overexpression increased the ATP levels by 55% and 63% in wild-type and necdin-null neurons, respectively (Fig. 5e). In necdin-null neurons, necdin increased the MTT and ATP levels to those similar to wild-type control levels.

We then examined whether necdin-null cortical neurons are susceptible to oligomycin, a mitochondrial ATP synthase inhibitor. Oligomycin significantly increased apoptosis of necdin-null neurons during the 4–24 h period (Fig. 5f), suggesting that endogenous necdin suppresses oligomycin-induced apoptosis of cortical neurons. In the LDH-based cytotoxicity assay, oligomycin increased the LDH levels by ∼50% in wild-type neurons and 2.3-fold in necdin-null neurons under LV-EmGFP-infected conditions (Fig. 5g), indicating that necdin-null neurons are highly susceptible to oligomycin. Remarkably, LV-mediated overexpression of necdin completely protected necdin-null neurons against oligomycin-induced damage. These results indicate that necdin promotes mitochondrial function to prevent oligomycin-induced degeneration of cortical neurons.

### Necdin prevents neurodegeneration in experimental PD models

**Figure 6:**
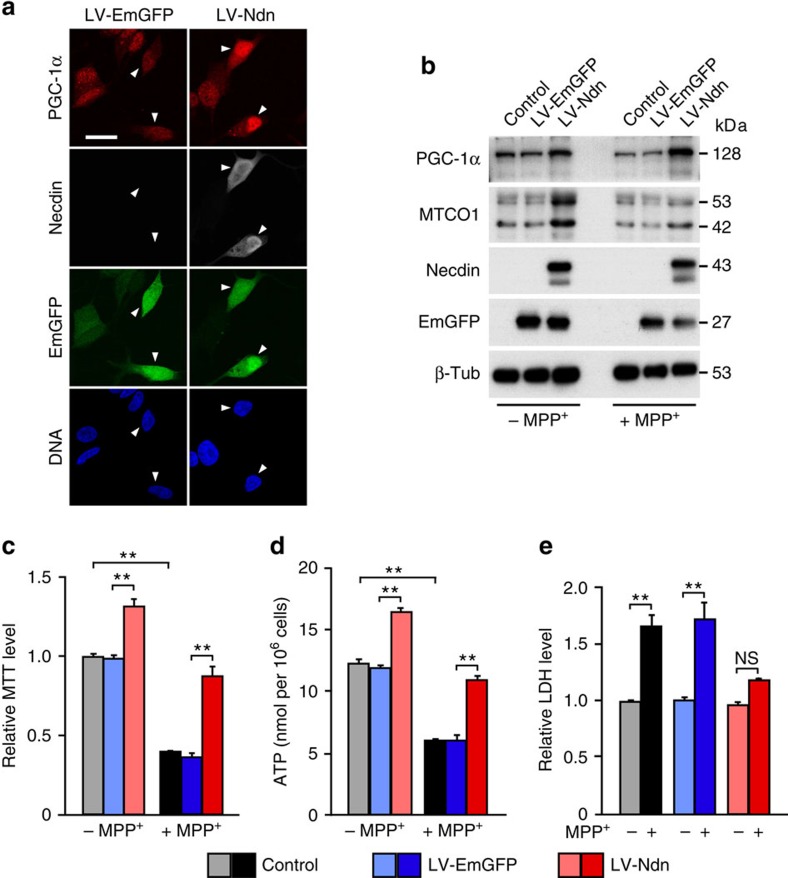
Necdin prevents MPP^+^-induced degeneration of human SH-SY5Y neuroblastoma cells. (**a**) SH-SY5Y cells were infected with LV expressing EmGFP (LV-GFP) or necdin (LV-Ndn), cultured in the presence of retinoic acid for 4 days for neural differentiation and triply immunostained for PGC-1α, necdin and EmGFP. Arrowheads, LV-infected cells. Scale bar, 10 μm. (**b**) LV-infected SH-SY5Y cells were treated without (–MPP^+^) or with 1 mM MPP^+^ (+MPP^+^) for 48 h. Expression of PGC-1α, MTCO1, necdin, GFP and β-tubulin (β-Tub) was analysed by western blotting. (**c**) LV-infected SH-SY5Y cells were treated with MTT for 4 h, and MTT-formazan deposits were measured by spectrophotometry (*n*=4). (**d**) ATP levels in LV-infected SH-SY5Y cells were analysed by chemiluminescence assay (*n*=3). (**e**) LDH levels released from LV-infected SH-SY5Y cells treated with 1 mM MPP^+^ for 48 h were quantified (*n*=4). Data represent means±s.e.m. **P<0.01, NS, not significant at *P*≥0.05; analysis of variance with Tukey–Kramer *post hoc* test.

The mitochondrial complex I inhibitor MPP^+^ (1-methyl-4-phenylpyridine) and its precursor MPTP (1-methyl-4-phenyl-1,2,3,6-tetrahydropyridine) selectively damage dopaminergic neurons and are commonly used for experimental PD models^18^. We first examined whether necdin prevents MPP^+^-induced degeneration of SH-SY5Y cells, a human neuroblastoma cell line susceptible to MPP^+^. LV-mediated overexpression of necdin increased the expression of PGC-1α and MTCO1 in SH-SY5Y cells as analysed by immunocytochemistry and western blotting (Fig. 6a,b). Necdin markedly increased the expression levels of PGC-1α 2.3- and 18.4-fold (compared with EmGFP controls) and those of MTCO1 9.3- and 8.6-fold (compared with EmGFP controls) in SH-SY5Y cells in the absence and presence of MPP^+^, respectively. Necdin increased the MTT levels 1.3- and 2.4-fold (compared with EmGFP controls) in the absence and presence of MPP^+^, respectively (Fig. 6c). Similarly, necdin increased the ATP levels in SH-SY5Y cells by 38% and 79% in the absence and presence of MPP^+^, respectively (Fig. 6d). In the cytotoxicity assay, MPP^+^ significantly increased the LDH levels in uninfected and LV-EmGFP-infected SH-SY5Y cells, whereas forced expression of necdin reduced the LDH levels near MPP^+^-untreated levels (Fig. 6e). These results suggest that necdin improves mitochondrial function to reduce MPP^+^-induced damage of SH-SY5Y cells.

**Figure 7:**
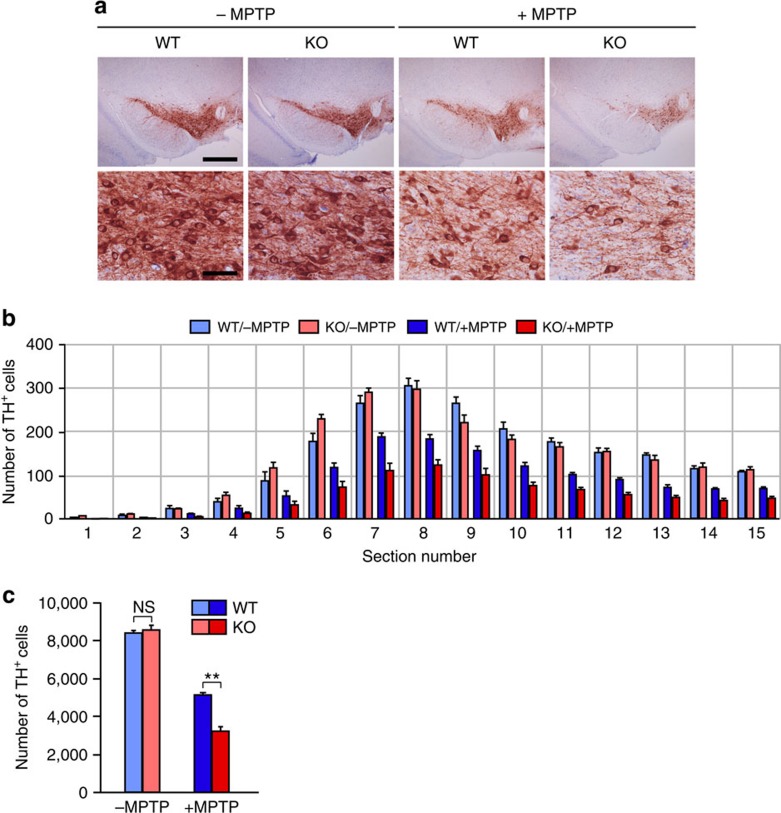
Necdin deficiency enhances MPTP-induced degeneration of dopaminergic neurons *in vivo*. (**a**) TH-expressing (TH^+^) neurons in the SN were analysed by immunohistochemistry (magnified in lower panels). Wild-type (WT) and necdin-null (knockout; KO) 13-week-old mice were injected intraperitoneally without (–MPTP) or with MPTP (+MPTP) for 5 consecutive days and killed 21 days after injection. Scale bars, 500 μm (upper) and 50 μm (lower). (**b**,**c**) SN sections were immunostained for TH, and the number of TH^+^ neurons in each section of the SNpc (**b**) and the total number (**c**) were presented (15 sections per mouse; *n*=4–5). Data represent means±s.e.m.; **P<0.01, NS, not significant at *P*≥0.05; analysis of variance with Tukey–Kramer *post hoc* test.

We next investigated whether necdin prevents MPTP-induced degeneration of dopaminergic neurons in the substantia nigra (SN) *in vivo* using necdin-null mice in the C57BL/6J background, which is susceptible to MPTP. Tyrosine hydroxylase-expressing (TH^+^) dopaminergic cells in the SN pars compacta (SNpc) were quantified after MPTP treatment (Fig. 7a and Supplementary Fig. 5a). There was no difference in the number of SNpc TH^+^ cells between wild-type and necdin-null mice without MPTP treatment, whereas SNpc TH^+^ cells in necdin-null mice were significantly reduced in number when treated with MPTP (Fig. 7b,c). The numbers of TH^+^ dopaminergic neurons were reduced throughout the SNpc area of necdin-null mice. This suggests that endogenous necdin in SNpc dopaminergic neurons is neuroprotective against MPTP toxicity.

Because necdin potentially prevents neuronal apoptosis through suppressing the E2F1-Cdc2 and p53-Bax axes^15^,^16^, we also analysed the mRNA levels of *necdin*, *E2F1*, *Cdc2* (*Cdk1*), *p53* and *Bax* in the SN of wild-type and necdin-null mice treated with MPTP (Supplementary Fig. 5b,c). However, there were no significant differences in the expression levels of these mRNAs between wild-type and necdin-null mice, suggesting that these pathways are not involved in MPTP-induced degeneration of nigral dopaminergic neurons seen in necdin-null mice.

**Figure 8:**
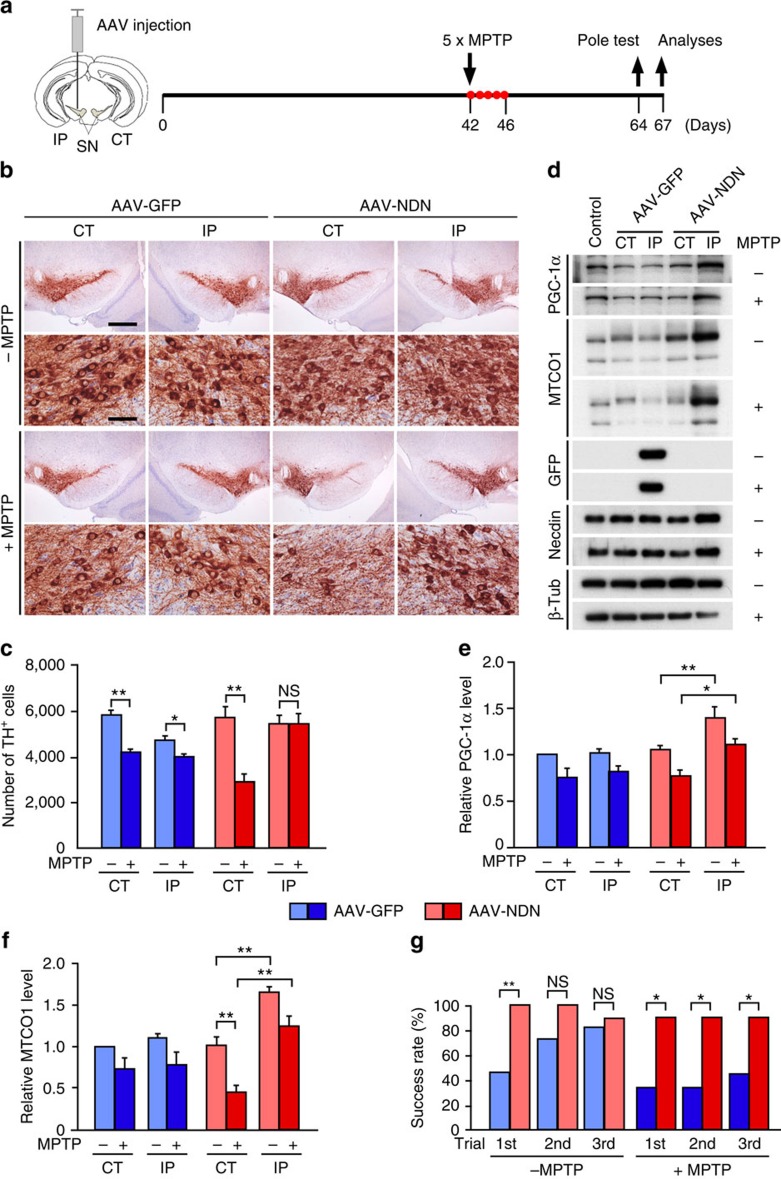
Necdin prevents MPTP-induced degeneration *in vivo* of dopaminergic neurons in experimental PD. (**a**) Schedule of AAV-mediated gene transfer into SN neurons and MPTP treatment. (**b**) TH^+^ dopaminergic neurons were analysed by immunohistochemistry (magnified in lower panels). AAV expressing GFP (AAV-GFP) or human necdin (AAV-NDN) was microinjected into the SN of 13-week-old mice and treated without (–MPTP) or with MPTP (+MPTP). CT, contralateral SN; IP, ipsilateral SN. Scale bars, 500 μm (top panel) and 50 μm (second panel). (**c**) SN sections were immunostained for TH, and TH^+^ neurons in the SNpc were counted (12 sections per mouse; *n*=3–5). (**d**) Expression of PGC-1α and MTCO1 in SN tissue extracts was analysed by western blotting. (**e**,**f**) Expression levels of PGC-1α (**e**) and MTCO1 (**f**) were quantified by densitometry and normalized to β-tubulin (β-Tub) levels (*n*=4–5). Data represent means±s.e.m.; *P<0.05, **P<0.01, NS, not significant at *P*≥0.05; analysis of variance with Tukey–Kramer *post hoc* test. (**g**) MPTP-induced behavioural changes of AAV-infected mice were assessed by the pole test. Success rates in three consecutive trials were presented (*n*=9–11). Data represent means; *P<0.05, **P<0.01, NS, not significant at *P*≥0.05; *χ*^2^-test.

To investigate whether necdin overexpression exerts protective effects on dopaminergic neurons *in vivo*, we used adeno-associated virus (AAV)-mediated gene delivery into the SN of MPTP-based PD model mice^24^,^25^ (Fig. 8a and Supplementary Fig. 6a). MPTP markedly reduced the number of TH^+^ neurons in the contralateral SNpc of mice injected with AAV expressing GFP (AAV-GFP), whereas injection with AAV expressing human *necdin* gene (AAV-NDN) fully protected TH^+^ neurons in the ipsilateral SNpc against MPTP-induced degeneration (Fig. 8b,c and Supplementary Fig. 6b,c). We then analysed the expression levels of PGC-1α and MTCO1 in the AAV-infected SN by western blotting (Fig. 8d). AAV-NDN infection significantly increased the necdin levels in the SN (relative necdin levels: MPTP-untreated, contralateral, 1.0±0.3, ipsilateral, 2.0±0.4; MPTP-treated, contralateral, 1.0±0.2, ipsilateral, 2.3±0.4, *P*<0.05; Student's *t*-test, *n*=4 per each group). AAV-mediated necdin overexpression increased the PGC-1α levels by 32% and 43% in the ipsilateral SN (compared with the contralateral SN levels) of untreated and MPTP-treated mice, respectively (Fig. 8e). Similarly, necdin increased the MTCO1 levels 1.6- and 2.8-fold in the ipsilateral SN of untreated and MPTP-treated mice, respectively (Fig. 8f). Furthermore, AAV-NDN-infected mice exhibited higher behavioural performance in the pole test than AAV-GFP-infected mice (Fig. 8g), indicating that necdin improves motor coordination of MPTP-treated mice. These results suggest that AAV-mediated necdin overexpression suppresses MPTP-induced degeneration of SN neurons *in vivo* by promoting PGC-1α-mediated mitochondrial biogenesis.

### Necdin deficiency promotes neurodegeneration

**Figure 9:**
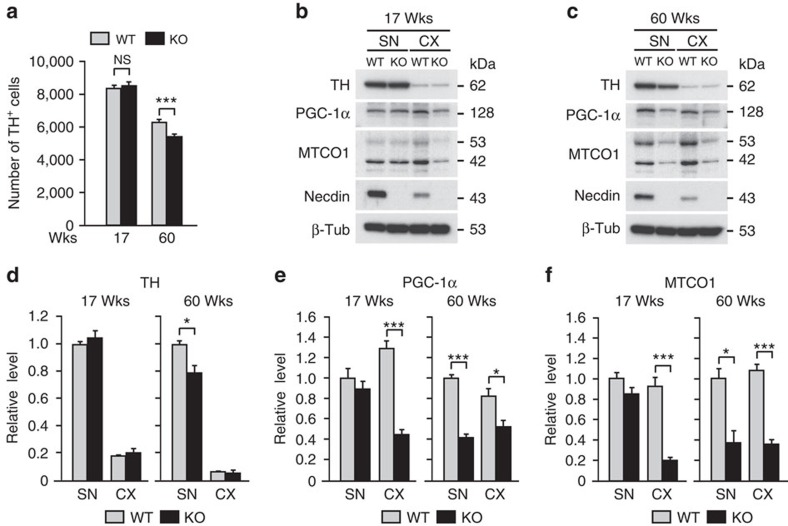
Necdin deficiency promotes degeneration of dopaminergic neurons in the SN of late adult mice. (**a**) TH^+^ neurons in the SN of wild-type (WT) and necdin-null (knockout; KO) mice at 17 and 60 weeks of age (wks) were analysed by immunohistochemistry. SN sections were immunostained for TH, and the total number of TH^+^ neurons were presented (15 sections per mouse; *n*=3–6). (**b**,**c**) Expression of TH, PGC-1α and MTCO1 in SN tissue extracts was analysed by western blotting. Durations of chemiluminescence detection were adjusted for quantification of each protein band in two age groups, and representative blot images of mice at 17 (**b**) and 60 weeks (**c**) are presented separately. (**d**–**f**) Expression levels of TH (**d**), PGC-1α (**e**) and MTCO1 (**f**) were quantified by densitometry of western blot signals and normalized to β-tubulin (β-Tub) levels (*n*=4 each). Values relative to the WT-SN level (=1) in each age group are shown. Data represent means±s.e.m.; *P<0.05, ***P<0.005; NS, not significant at *P*>0.05; Student's *t*-test.

We investigated the neuroprotective effect of endogenous necdin on SN dopaminergic neurons using necdin-null mice. We first analysed the number of TH^+^ neurons in the SNpc of wild-type and necdin-null mice at their adult (17 weeks old) and late adult (60 weeks old) stages (Fig. 9a). Although there was no significant difference in the number of SNpc TH^+^ cells between wild-type and necdin-null mice at 17 weeks of age, the number of SNpc TH^+^ cells was significantly reduced by 15% in necdin-null mice at 60 weeks of age. We then analysed the expression levels of TH, PGC-1α and MTCO1 in the SN by western blotting (Fig. 9b,c). Similar to the results of TH^+^ neuron counting, the expression level of TH was decreased by 21% in the SNpc of necdin-null mice 60 weeks old (Fig. 9d). Furthermore, the expression levels of PGC-1α and MTCO1 in the SN decreased by 58% and 63%, respectively, in necdin-null mice 60 weeks old, whereas there were no differences in the SN levels of PGC-1α and MTCO1 between wild-type and necdin-null mice 17 weeks old (Fig. 9e,f). On the other hand, PGC-1α and MTCO1 levels in the cortex of necdin-null mice decreased at 17 weeks of age by 66% and 78%, respectively, and at 60 weeks of age by 37% and 67%, respectively. These results suggest that endogenous necdin prevents degeneration of nigral dopaminergic neurons by maintaining the level of mitochondrial biosynthesis.

## Discussion

The present study has shown that necdin upregulates the expression of neuronal PGC-1α at the post-translational level. In primary cortical neurons and developing brain tissues, the levels of necdin parallel with those of PGC-1α and the mitochondrial DNA-encoded protein MTCO1. In addition, PGC-1α and MTCO1 levels in the cerebral cortex *in vivo* are much lower in necdin-null mice than in wild-type mice at adult stages. These results suggest that necdin is an intrinsic positive regulator of PGC-1α-mediated mitochondrial biogenesis. Virus vector-mediated overexpression of necdin in primary cortical neurons and nigral dopaminergic neurons *in vivo* upregulates the expression of PGC-1α and MTCO1, indicating that endogenous expression levels of those proteins are controllable by exogenous necdin. Thus, necdin is likely to act as a molecular rheostat to control neuronal mitochondrial biogenesis via PGC-1α. We speculate that necdin and PGC-1α, both of which are expressed in virtually all neurons, cooperate to maintain high mitochondrial activities required for neuronal function and survival.

We have employed the mitochondrial oxidative phosphorylation inhibitors in combination with viral vector-mediated necdin gene delivery to demonstrate the neuroprotective effects of necdin. The combination of primary cortical neurons and the ATP synthase inhibitor oligomycin showed that necdin-null neurons are highly susceptible to oligomycin-induced apoptosis, which is fully prevented by overexpression of necdin. To investigate the effects of necdin on neurodegeneration *in vivo*, we have selected nigral dopaminergic neurons, which are highly susceptible to the mitochondrial complex I inhibitor MPTP, a lipophilic protoxin metabolized in glial cells to MPP^+^, which is concentrated in dopaminergic neurons via the dopamine transporter^18^. Before the analysis of *in vivo* PD model, we confirmed the toxic effect of the active MPTP metabolite MPP^+^ on SH-SY5Y neuroblastoma cells, a human catecholaminergic cell line that exhibits apoptotic changes in response to MPP^+^ (refs 26, 27). Necdin-mediated protection of SH-SY5Y cells against MPP^+^ toxicity led to further demonstrate the neuroprotective effect of necdin on nigral dopaminergic neurons *in vivo* against MPTP-induced degeneration. These findings implicate that mitochondrial dysfunction-induced neurodegeneration is preventable by enhancing neuronal mitochondrial biogenesis through overexpression of necdin.

The present study clarified that necdin strongly stabilizes PGC-1α by inhibiting its degradation in the ubiquitin-proteasome system. Necdin interacted with PGC-1α via the N-terminal region that contains the PEST (proline, glutamic acid, serine, threonine) sequence, a domain critical for ubiquitin-proteasomal degradation of PGC-1α in the nucleus^28^,^29^. Rnf34 (ref. 22), Fbxw7 (ref. 23) and RNF2 (ref. 30) act as PGC-1α E3 ubiquitin ligases. Necdin efficiently suppressed Rnf34- and Fbxw7-mediated ubiquitination of PGC-1α. We speculate that necdin forms a stable nuclear complex with PGC-1α in neurons and protects PGC-1α from ubiquitination mediated by these E3 ubiquitin ligases. The N-terminal amino-acid sequences of PGC-1α and PGC-1β are well conserved^5^, and these PGC-1 family proteins increase mitochondrial density in primary cortical neurons^21^. Thus, we infer that necdin protects PGC-1α and PGC-1β against proteasomal degradation to promote their effects on mitochondrial biogenesis in mammalian neurons.

A genome-wide expression study revealed that PGC-1α-regulated genes controlling cellular bioenergetics are underexpressed in SN neurons affected by PD^31^. PGC-1α-null mutant mice exhibit striatal lesions^32^, and their SN neurons are highly susceptible to MPTP^33^. Moreover, PGC-1α overexpression protects SN neurons against neurodegenerative insults^34^,^35^. These findings imply that PGC-1α contributes to the resistance of SN dopaminergic neurons against PD-associated neurodegeneration. In contrast to the beneficial effects of PGC-1α, sustained overexpression of PGC-1α in the SN induces degeneration of dopaminergic neurons^36^,^37^. These inconsistent results suggest that control of exogenous PGC-1α expression levels is crucial for the neuroprotective effects. In contrast, AAV-mediated overexpression of necdin increases endogenous PGC-1α protein levels by inhibiting its ubiquitin-proteasomal degradation at the post-translational level. Thus, we speculate that the *necdin* gene delivery increases the PGC-1α levels in dopaminergic neurons within a physiologically tolerable extent that is sufficient for effective neuroprotection.

Recombinant AAV displays efficient transduction of SN dopaminergic neurons *in vivo* and has been often used for analysing the pathogenic mechanism of PD and developing therapeutic strategies. For example, AAV-mediated overexpression of α-synuclein, a major structural component of Lewy bodies seen in neurons undergoing degeneration in PD, induces PD-like neurodegeneration^38^. AAV-mediated overexpression of Parkin, a ubiquitin E3 ligase whose mutations cause recessively inherited early-onset PD^39^, ameliorates α-synuclein-mediated or MPTP-induced degeneration of dopaminergic neurons^25^,^40^. Moreover, AAV-mediated delivery of PARIS (also known as ZNF746), a substrate of Parkin, induces selective dopaminergic neuron loss, which is prevented by AAV-mediated overexpression of Parkin^34^. Gene therapy for PD using AAV-mediated gene delivery has reached human clinical trials^41^. Thus, we propose that AAV-mediated *necdin* gene delivery provides a novel strategy for mitochondrial biogenesis-based neuroprotection in PD.

Human *necdin* gene (*NDN*) is located on chromosome 15q11–q12, and its expression is absent in neurons affected by Prader–Willi syndrome (PWS), a classic genomic imprinting-associated neurodevelopmental disorder^11^,^12^. The present study has shown that the brain levels of mitochondrial proteins and mitochondrial activities are significantly reduced in necdin-null neurons. These findings raise the possibility that necdin-null neurons affected in PWS have low mitochondrial activities and reduced ATP levels during neuronal development. Motor proteins such as myosin, kinesin and dynein families are powered by ATP hydrolysis and play key roles in neuronal morphogenesis and network formation during brain development^42^. Mice carrying mutated paternal *Ndn* exhibit a variety of neurodevelopmental abnormalities such as reduced neuron number, impaired neuronal migration and abnormal axon extension in the embryonic brain^43^,^44^,^45^,^46^,^47^,^48^,^49^. Although there is limited information about the neuropathological abnormalities in the PWS-affected brain, earlier studies using magnetic resonance imaging have revealed multiple morphological abnormalities such as ventriculomegaly, reduced sizes of specific areas and differences in grey and white matter volumes in the brain of patients with PWS^50^,^51^,^52^. Thus, we speculate that reduced mitochondrial activities in necdin-null neurons contribute, at least in part, to these neurodevelopmental abnormalities seen in PWS.

Our findings suggest that necdin and PGC-1α cooperate to promote mitochondrial biogenesis in various types of neurons and prevent mitochondria-associated neurodegeneration. Necdin-null mice exhibit low expression of the PGC-1α protein in the brain, where the number of nigral dopaminergic neurons is significantly reduced in late adulthood. Necdin is abundantly expressed in spinal cord motor neurons^10^, and their mitochondrial dysfunction causes ALS^53^. PGC-1α is also suggested to contribute to the pathogeneses of ALS^54^. Intriguingly, necdin expression in spinal cord motor neurons of SOD1 G93A mutant mice, an SOD1 gene mutant ALS model, increases significantly at the early presymptomatic stage and decreases at the late symptomatic stage^55^. We speculate that necdin expression is upregulated for neuroprotection at the early stage of neurodegeneration but declines at the advanced stage. A multi-cohort transcriptional meta-analysis has revealed that necdin expression is specifically diminished in major human neurodegenerative diseases^56^. These findings raise the possibility that necdin is involved in neuronal resistance or resilience against neurodegenerative insults. Thus, gene therapy using virus vector-mediated necdin gene delivery into specific neurons at risk will be a promising avenue for prevention or therapeutic intervention of neurodegenerative diseases. The present findings also warrant further studies on the neuron-specific mechanism of mitochondrial biogenesis and its association with neurodegenerative diseases.

## Methods

### *Ndn* knockout mice

*Ndn* knockout mice (*Ndn*^*tm/Ky*^) were generated and maintained as described^46^. Heterozygous male mice (*Ndn*^+m/−p^) (>25 generations on the ICR background and 20 generations on the C57BL/6J background) were crossed with wild-type (*Ndn*^+m/+p^) female mice to obtain *Ndn*^+m/+p^ and *Ndn* knockout (*Ndn*^+m/-p^) littermates. Genotypes of all mice were analysed by PCR for mutated *Ndn* locus. C57BL/6J mice were used for demonstrating MPTP-induced neurodegeneration and phenotypes of *Ndn* knockout mice (13, 17 and 60 weeks of age). The study was approved by the Animal Experiment Committee (Approval No. 24-04-0) and Recombinant DNA Committee (Approval No. 3642) of Institute for Protein Research, Osaka University, and were performed in accordance with national, institutional and the ARRIVE guidelines.

### Cell cultures

Primary cortical neurons were prepared from the forebrain of ICR mice (Japan SLC) at E14.5 as described^16^. The cortex was dissected and incubated in 0.5 ml of Ca^2+^/Mg^2+^-free Hanks' balanced salt solution with 0.05% trypsin for 5 min at 37 °C. Tissues were dissociated with 10% fetal bovine serum (FBS) in Dulbecco's modified eagle medium (DMEM) and centrifuged at 200*g* for 3 min. Pellets were resuspended in Neurobasal medium (Life Technologies) supplemented with 2 mM L-glutamine, kanamycin/penicillin and B-27 supplement (1:50 dilution, Life Technologies), plated at a density of ∼1 × 10^5^ cells per cm^2^ in culture dishes, and incubated for 4 days *in vitro* (4 DIV) before analyses. For preparation of primary NPCs, dissociated cortical cells were cultured as floating neurospheres in DMEM/F12 (Life Technologies) supplemented with B-27 (1:50 dilution), 20 ng ml^−1^ epidermal growth factor (PeproTech) and 20 ng ml^−1^ basic fibroblast growth factor (PeproTech) for 48 h at 37 °C under humidified 5% CO_2_ conditions. HEK293A cells (Life Technologies) and SH-SY5Y cells (gift from Dr June Biedler, Memorial Sloan-Kettering Cancer Center)^26^ were cultured in DMEM containing 10% FBS at 37 °C under humidified 5% CO2 conditions.

### DNA microarray

Total RNA was extracted from primary cortical neurons at 4 DIV, with phenol and guanidine thiocyanate mixture (TRI Reagent, Molecular Research Center). Expression profiling was performed using CodeLink Mouse Whole Genome Bioarray (Applied Microarrays) according to the manufacturer's protocol. Total RNA (1 μg) was labelled and used for microarray. Array slides were hybridized at 37 °C for 18 h, washed, dried and scanned with a microarray scanner (GenePix 4400A, Molecular Devices). The signal intensities were quantified using CodeLink Expression Analysis Software v5.0 (Applied Microarrays). Gene ontology and clustering of microarray data were analysed using Microarray Data Analysis Tool Ver3.2 (Filgen) and Cluster and TreeView programmes^57^.

### Quantitative reverse transcription–PCR

Total RNA was extracted with the guanidine thiocyanate mixture, and contaminating DNA was digested with RQ1 RNase-free DNase (Promega). For qRT–PCR, total RNA (2 μg) was reverse-transcribed to cDNA using Transcriptor First Strand cDNA Synthesis Kit (Roche Diagnostics). cDNA (10 ng) was used as templates for PCR mixture (LightCycler FastStart DNA Master^PLUS^ SYBR Green I, Roche Diagnostics) in a real-time PCR system (LightCycler 1.5, Roche Diagnostics). Primers for qRT–PCR are described in Supplementary Table 2.

### Mitochondrial DNA/mass measurements

Total genomic DNA was extracted from cortical neurons at 4 DIV, and mitochondrial DNA content was quantified by real-time PCR with LightCycler 1.5 using *D-loop* primers (forward, 5′- GGTTCTTACTTCAGGGCCATCA-3′; reverse, 5′-GATTAGACCCGTTACCATCGAGAT-3′). Ribosomal protein P0 gene *Rplp0* (*R36B4*) primers (forward, 5′-GTGGGTAATCTCACTGGAAAG-3′; reverse, 5′-TTGTCCCAGACTAGCTATGG-3′) were used as a nuclear genome control for normalizing the *D-loop* level. For determination of mitochondrial mass in neurons, cortical neurons at 4 DIV were incubated with 100 nM MitoTracker Green FM (Life Technologies) at 37 °C for 30 min, and analysed by flow cytometry with FACSCalibur (BD Biosciences).

### Western blot analysis

Cells or tissues were homogenized with lysis buffer containing 10 mM Tris-HCl, pH 8.0, 150 mM NaCl, 1 mM EDTA, 1% IGEPAL CA-630 (MP Biomedicals), 10% glycerol and protease inhibitor cocktails (Complete, Roche Diagnostics). The protein concentration was determined by the Bradford method (Bio-Rad). Proteins (2–10 μg per lane) were separated by 10% SDS–PAGE and electroblotted to polyvinylidene difluoride membranes (Immobilon, Merck Millipore). The membranes were incubated with primary antibodies against the mitochondrial complex I–V subunits NDUFB8, SDHB, UQCRC2, MTCO1 and ATP5A (total OXPHOS rodent WB antibody cocktail of antibodies, ab110413, 1:500, Abcam), necdin (NC243, 1:3,000) (ref. 58), β-tubulin (TUB2.1, 1:1,000, Sigma-Aldrich), PGC-1α (1:500, PGCAN), γ-tubulin (GTU-88, 1:1,000, Sigma-Aldrich), nestin (ST-1, 1:500) (ref. 59), MAP2 (1:5,000, gift from Dr Michio Niinobe, Osaka University), actin (JLA20, 1:1,000, Sigma-Aldrich), MTCO1 (ab14705, 1:200, Abcam), LC3 (8E10, 1:1,000, MBL), PCNA (PC10, 1:1,000, Santa Cruz Biotechnologies), Myc (9E10, 1:10), V5 (R960-25, 1:5,000, Life Technologies), FLAG (M2, 1:500, Sigma-Aldrich) and GFP (JFP-J1, 1:200, Riken Cell Bank). Anti-PGC-1α antibody (PGCAN) was generated in rabbit against a purified recombinant protein of maltose-binding protein (MBP) fused to N-terminal PGC-1α (amino acids 1–120). Characterization of PGCAN is depicted in Supplementary Fig. 1. After incubation with peroxidase-conjugated IgGs (Cappel), proteins were detected by chemiluminescence method (Chemiluminescence Reagent Plus, PerkinElmer). Signal intensities were quantified with ImageJ 1.44 software. Images have been cropped for presentation. Full-size images are presented in Supplementary Fig. 7.

### MTT assay

Mitochondrial MTT (3-[4,5-dimethylthiazol-2-yl]-2,5 diphenyl tetrazolium bromide) metabolic activity in primary cortical neurons and differentiated SH-SY5Y cells was analysed by Cell Proliferation Kit I (MTT, Roche Diagnostics) according to the manufacturer's protocol. For detection of intracellular MTT-formazan deposits, primary neurons were plated on a 35-mm imaging dish (μ-Dish^35 mm, high^, ibidi) precoated with poly-L-ornithine (Sigma-Aldrich), and treated with 1.1 mM MTT. Images were observed with a differential interference contrast microscope (IX70, Olympus). For quantification of MTT metabolic activity, cells were plated on 96-well dishes (BioLite 96-well Multidish, Thermo Scientific) at 5 × 10^4^ cells, and treated with 1.1 mM MTT for 4 h and lysed for 12 h in the lysis buffer containing 10% SDS. MTT-formazan deposits were solubilized, measured by spectrophotometry as optical density (OD) at wavelength 550/690 nm (OD 550/690).

### LDH assay

LDH levels released from damaged cells were measured using Cytotoxicity Detection kit (LDH, Roche Diagnostics) according to the manufacturer's protocol. Culture medium of differentiated neurons was centrifuged at 200*g* for 5 min. The supernatant was collected, incubated with the LDH reaction mixture at room temperature for 30 min, and measured by spectrophotometry at a dual wavelength of 492/690 nm.

### Complex I activity assay

Complex I activity was measured using an assay kit based on the immunocapture with immobilized anti-Complex I antibodies combined with in-gel activity measurement (dipstick assay, ab109720, Abcam). Cell lysates (30 μg protein) were used for the assay according to the manufacturer's protocol. Complex I activity was measured by incubating with NADH and nitrotetrazolium blue. Signals of resulting blue-purple precipitates on the dipstick were analysed by densitometry and quantified using ImageJ 1.44 software.

### ATP assay

ATP levels in cell lysates were analysed by luciferase chemiluminescence-based assays (CellTiter-Glo Luminescent Cell Viability Assay kit, Promega) according to the manufacturer's protocols. Briefly, cell lysates were mixed with CellTiter-Glo Reagent for 2 min by shaking vigorously and settled for 10 min. Chemiluminescence in reaction mixtures was measured with a luminometer (Lumat LB9501, Berthold).

### Mitochondrial membrane potential assay

For determination of mitochondrial membrane potential in neurons, cortical neurons at 4 DIV were incubated with 100 nM chloromethyl-X-rosamine (MitoTracker Red, Life Technologies) at 37 °C for 30 min, and analysed by flow cytometry with FACSCalibur (BD Biosciences).

### Immunohistochemistry

Brain tissues of mouse embryos at E14.5 were fixed with 4% paraformaldehyde in phosphate buffer (pH 7.4) overnight and cryoprotected by immersion in 20% sucrose overnight. Frozen 12-μm-thick tissue sections were incubated with primary antibodies at 4 °C overnight and fluorescence dye-conjugated secondary antibodies at room temperature for 60 min. The primary antibodies used are rabbit polyclonal antibody against PGC-1α (PGCAN, 1:500), MAP2 (1:1,000), necdin (NC243, 1:500), guinea pig polyclonal antibody against necdin (GN1, 1:500) (ref. 46) and mouse monoclonal antibodies against Sox2 (245610, 1:300, R and D Systems), βIII-tubulin (1:1,000, Promega) and MTCO1 (ab14705, 1:1,000). The secondary antibodies used are Alexa488-conjugated anti-rabbit IgG (1:1,000, Molecular Probes), Alexa555-conjugated anti-guinea pig IgG (1:1,000, Molecular Probes) and Alexa488-conjugated anti-mouse IgG (1:1,000, Molecular Probes). Nuclear DNA was counterstained with 3.3 μM Hoechst 33342 (Sigma-Aldrich). Immunofluorescence images were observed with a fluorescence microscope (BX51, Olympus), taken by CCD (charge-coupled device) camera system (DP73, Olympus), and processed using Adobe PhotoShop CS4 software. For immunostaining of adult mouse brain sections, free-floating sections were incubated with a rabbit polyclonal antibody to TH (1:5,000, Calbiochem) in PBS containing 0.05% Triton X-100 (PBST) and 2% blocking reagent (Block Ace, Sumitomo Dainippon Pharma) at 4 °C for 48 h, with biotinylated anti-rabbit IgG secondary antibody (1:500, Vector Laboratories) in PBST containing 2% Block Ace for 2 h, and with avidin–biotin–peroxidase complex (ABC Elite, Vector Laboratories) for 1 h. After reaction, sections were treated with 0.04% diaminobenzidine in 50 mM Tris-HCl (pH 7.6) and 0.02% hydrogen peroxide with 0.04% nickel chloride. Images were observed with a light microscope (BZ-9000, Keyence) and processed using Adobe PhotoShop CS4 software.

### Immunocytochemistry

Cells were fixed with 10% formalin solution at room temperature for 20 min and permeabilized with methanol at room temperature for 20 min. Fixed cells were incubated with primary antibodies at 4 °C overnight, and with secondary antibodies at room temperature for 90 min. Primary antibodies used for immunocytochemistry are rabbit polyclonal antibody against PGC-1α (PGCAN, 1:500), guinea pig polyclonal antibody against necdin (GN1, 1:500), mouse monoclonal antibody against Myc (9E10, 1:10) and rat monoclonal GFP (JFP-J1, 1:200). The secondary antibodies Alexa488-conjugated anti-rabbit, anti-mouse and anti-rat IgGs (1:1,000, Molecular Probes), and Alexa555- and Alexa633-conjugated anti-guinea pig IgG (1:1,000, Molecular Probes) were used. Nuclear DNA was counterstained with 3.3 μM Hoechst 33342 (Sigma-Aldrich). Images were observed with a fluorescence microscope (BX51, Olympus) and confocal laser-scanning microscope (FV1000 BX61, Olympus), taken by CCD camera system (DP73, Olympus), and processed using Adobe PhotoShop CS4 software.

### Fluorescence microphotometry

Cortical sections or transfected cells were immunostained for PGC-1α and MTCO1. Signal intensities were quantified by fluorescence microphotometry as described^17^. Fluorescence images were captured with a CCD image sensor (CoolSNAP monochrome, Nippon Roper) as 12-bit digital monochrome images. Fluorescence intensities of PGC-1α and MTCO1 were analysed using fluorescence image analysis software (Lumina Vision, Mitani).

### Co-immunoprecipitation assays

For detection of endogenous binding between necdin and PGC-1α, lysates of mouse embryonic forebrain (1 mg) were incubated with guinea pig anti-necdin antibody (GN1, 1:100). Bound proteins were pelleted with Protein A-Sepharose (nProtein A Sepharose 4 Fast Flow, GE Healthcare), separated by 10% SDS–PAGE and detected by western blotting. For detection of binding between necdin and PGC-1α in transfected cells, HEK293A cells were transfected with combinations of expression vectors by the calcium phosphate method and collected after 24 h. Cell lysates (200 μg) were incubated with antibodies at 4 °C for 2 h, pelleted with Protein A-Sepharose, separated by 10% SDS–PAGE and detected by western blotting. PGC-1α expression vector was constructed by subcloning cDNA encoding full-length mouse PGC-1α and PGC-1β (gifts from Dr Akira Kakizuka, Kyoto University) into pcDNA3.1+ and 6Myc-tagged pcDNA3.1+. cDNAs encoding PGC-1α deletion mutants of amino acids 1–200 (N-terminal), 201–400 (transcriptional repression), 401–550 (intermediate) and 551–797 (C-terminal) were generated using synthetic oligonucleotide primers and subcloned into the expression vector.

### Co-transfection assays

HEK293A cells were transfected with combinations of expression vectors by the calcium phosphate method and collected after 24 h. For CHX treatment, transfected HEK293A cells were incubated with DMEM/10% FBS containing 500 μM CHX (Sigma-Aldrich) for 60 min. HEK293A cells were transfected with combinations of cDNAs and treated with 10 μM MG132 (Peptide Institute) for 3 h before harvest. For PGC-1α ubiquitination assay, full-length cDNAs encoding the PGC-1α E3 ubiquitin ligases Rnf34 (NM_030564) and Fbxw7 (NM_080428) were cloned from E14.5 mouse forebrain cDNAs used as a PCR template, sequenced, attached with the V5-encoding sequence at the 3′-end, and subcloned into pcDNA3.1+. Proteins were immunoprecipitated with anti-PGC-1α antibody (PGCAN,1:50) and analysed by western blotting.

### *In vitro* binding analysis

Deletion mutants of PGC-1α were subcloned into pMAL-C2 vector to make MBP fusion proteins. MBP-fused mutant proteins were affinity purified with amylose resin, and incubated with His-tagged necdin (200 ng) at 4 °C for 30 min in 0.5 ml of the binding buffer containing 20 mM Tris-HCl, pH7.5, 200 mM NaCl and 1 mM EDTA as described^16^. After washing, bound His-tagged necdin was eluted with 20 mM maltose and detected by western blotting with anti-necdin antibody. MBP fusion proteins were detected by Coomassie Brilliant Blue staining.

### Viral vectors

Recombinant LVs were produced in HEK293FT cells by transfecting SIN vector plasmids and two or three helper plasmids using calcium phosphate method as described^59^. Necdin and EmGFP cDNAs were subcloned into pENTR1A entry vector (Life Technologies) to construct the destination vectors CSII-EF1α-necdin-IRES-EmGFP and CSII-EF1α-IRES-EmGFP to make LV-Ndn and LV-EmGFP, respectively. EmGFP (Life Technologies) was used for an expression indicator and negative control for necdin overexpression. The viral titre was measured by serial dilution on HEK293FT cells and determined as GFP-positive cells by fluorescence-activated cell sorting analysis. For AAV serotype-1 vector preparation, pAAV-MCS carrying cytomegalovirus promoter (Stratagene) carrying human necdin cDNA (NM_002487) and humanized GFP were used to make AAV-NDN and AAV-GFP, respectively, as described^25^. For high-titre viral stocks, AAV vectors were purified by ultracentrifugation in a density gradient with OptiPrep (Axis-Shield PoC AS), which was removed by ultrafiltration using Centricon Plus-20 (10,000 molecular weight cut-off, Millipore). Averaged titres of AAV-NDN and AAV-GFP were 1 × 10^12^ genomes per ml.

### Oligomycin-induced neurotoxicity assay

Primary cortical neurons were cultured for 4 days and treated with 20 μg ml^−1^ oligomycin (Sigma-Aldrich). For quantifying apoptotic neurons, cultures were stained with 3.3 μM Hoechst 33342 (Sigma-Aldrich) for 5 min before fixation, and cortical neurons carrying condensed or fragmented nuclei were counted. For LV infection, primary cortical cells were infected with LVs at multiplicity of infection (m.o.i.) of 2, incubated in DMEM/F12-based medium containing 20 ng ml^−1^ epidermal growth factor and 20 ng ml^−1^ basic fibroblast growth factor for 30 min, and cultured for 4 days in Neurobasal medium for neuronal differentiation. Mean viral infection efficiency was more than 92%. For LDH release assay, neurons were treated with oligomycin for 6 h.

### MPP^+^-induced cytotoxicity assay

LV vectors were infected into undifferentiated SH-SY5Y cells (gift from Dr June Biedler, Memorial Sloan-Kettering Cancer Center) at m.o.i. 2. Mean viral infection efficiency was 88%. Infected cells were incubated in Neurobasal medium containing 10 μM retinoic acid (Sigma-Aldrich) for 4 days before analyses. Infected SH-SY5Y cells were treated with 1 mM MPP^+^ (Sigma-Aldrich) for 48 h.

### MPTP-induced neurodegeneration analysis

In necdin-null mice of C57BL/6J background, 13-week-old mice were treated with MPTP-HCl (30 mg per kg body weight per day, Sigma-Aldrich) dissolved in saline for 5 consecutive days. Control mice without MPTP treatment were injected with saline. AAV vectors were stereotaxically injected into the SN of 13-week-old male C57BL/6J mice (Japan SLC) as described^25^. AAV-infected mice were injected intraperitoneally 42 days after AAV infection, with MPTP for 5 consecutive days. Control mice without MPTP treatment were injected with saline. MPTP-treated mice were killed 21 days after the last injection of MPTP. MPTP was handled in accordance with the guidelines^60^. For quantifying TH-expressing (TH^+^) cells, coronal 20-μm-thick brain sections were cut serially using a cryostat (CM1900, Leica Microsystems). Sections were stained with anti-TH antibody and counterstained with Nissl. TH- and Nissl-double-positive neurons in the SNpc were analysed by unbiased stereological counting method^25^. Cells having optimally visualized nuclei and nucleoli were counted to avoid double counting. TH^+^ cells in every fourth 20-μm section were counted so that 15 sections (total 60 sections for ∼1.2 mm) cover the entire SNpc extent. For western blotting, brain blocks including the entire SN were cut coronally at 2-mm thickness. A ventral part of the midbrain including the SN (∼1.2 mm from the ventral end) was dissected horizontally, and immediately frozen in liquid nitrogen for tissue extraction. Pole test was performed 3 days before western blot analysis according to the method described^61^ at 20:00. Mice were placed on the top of a 48-cm-long 1-cm-diameter wooden rod. Mice performed three trials with 30-s intervals, and success rates to reach the floor within 80 s were measured.

### Statistics

Statistical significance was tested using an unpaired Student's *t*-test, one-way analysis of variance followed by Tukey–Kramer *post hoc* test, or *χ*^2^-test. A significance of *P*<0.05 was required for rejection of the null hypothesis.

## Additional information

**Accession codes:** Microarray data have been deposited to the Gene Expression Omnibus (GEO) under the accession number GSE63498.

**How to cite this article:** Hasegawa, K. *et al.* Promotion of mitochondrial biogenesis by necdin protects neurons against mitochondrial insults. *Nat. Commun.* 7:10943 doi: 10.1038/ncomms10943 (2016).

## Supplementary Material

Supplementary Information Supplementary Figures 1-7 and Supplementary Tables 1-2.
