# Supplementary material for: Promotion of mitochondrial biogenesis by necdin protects neurons against mitochondrial insults
Source: Nat Commun. 2016 Mar 14;7:10943. doi: 10.1038/ncomms10943 (PMC4793078; doi:10.1038/ncomms10943)
Supplement: Supplementary Information — Supplementary Figures 1-7 and Supplementary Tables 1-2. [file ncomms10943-s1.pdf]

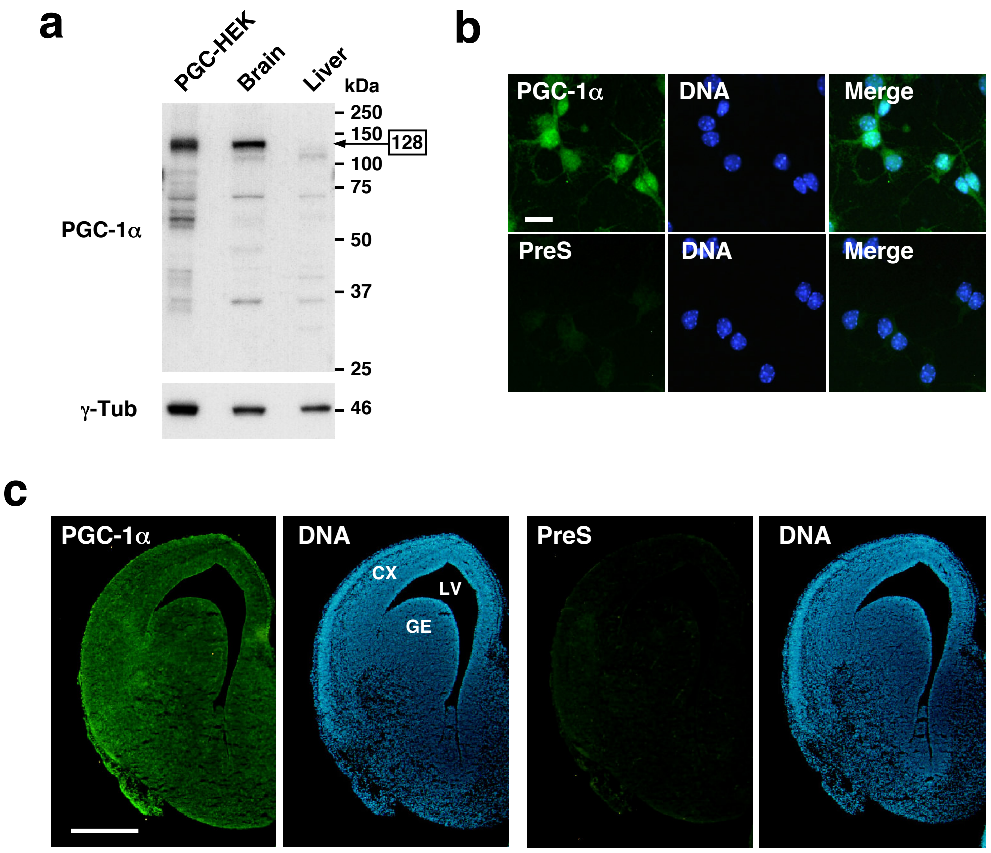

Supplementary Figure 1 | Characterization of a new antibody against mouse PGC-1α.
(**a**) Anti-PGC-1α antibody (PGCAN) was raised in rabbit against a purified recombinant protein of maltose binding protein fused to PGC-1α N-terminal region (amino acids 1-120). Expression of PGC-1α in PGC-1α cDNA-transfected HEK293A cells (PGC-HEK), brain, and liver of E14.5 mice was analyzed by Western blotting with PGCAN. γ-Tub, γ-tubulin (loading control). (**b**) PGC-1α in primary cortical neurons was detected by immunocytochemistry using PGCAN (upper) and preimmune serum (PreS)(lower). DNA was counterstained with Hoechst 33342. Scale bar, 10 μm. (**c**) PGC-1α in the E14.5 mouse forebrain was detected by immunohistochemistry using PGCAN (left) and PreS (right). CX, cortex; GE, ganglionic eminence; LV, lateral ventricle. Scale bar, 500 μm.

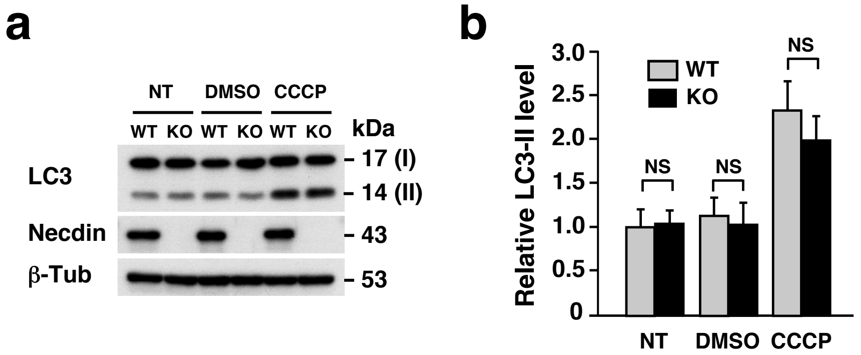

Supplementary Figure 2 | Necdin does not affect mitochondrial degradation by CCCP-induced mitochondrial uncoupling. (**a**) Expression of LC3, necdin, and β-tubulin (β-Tub) in cortical neurons treated with CCCP. Primary cortical neurons prepared from E14.5 mouse cortex of wild-type (WT) and necdin-null (KO) mice were cultured for 4 days and treated with 10 μM CCCP for 24 h according to the method of Cai et al. [19]. Expression of LC3, necdin, and β-tubulin (β-Tub) was analyzed by Western blotting. NT, non-treated control; DMSO, vehicle control. (**b**) LC3-II expression levels. Expression levels of LC3-II were quantified by densitometry. Data represent means ± s.e.m. (n = 4); NS, $P \geq 0.05$; Student's t test.

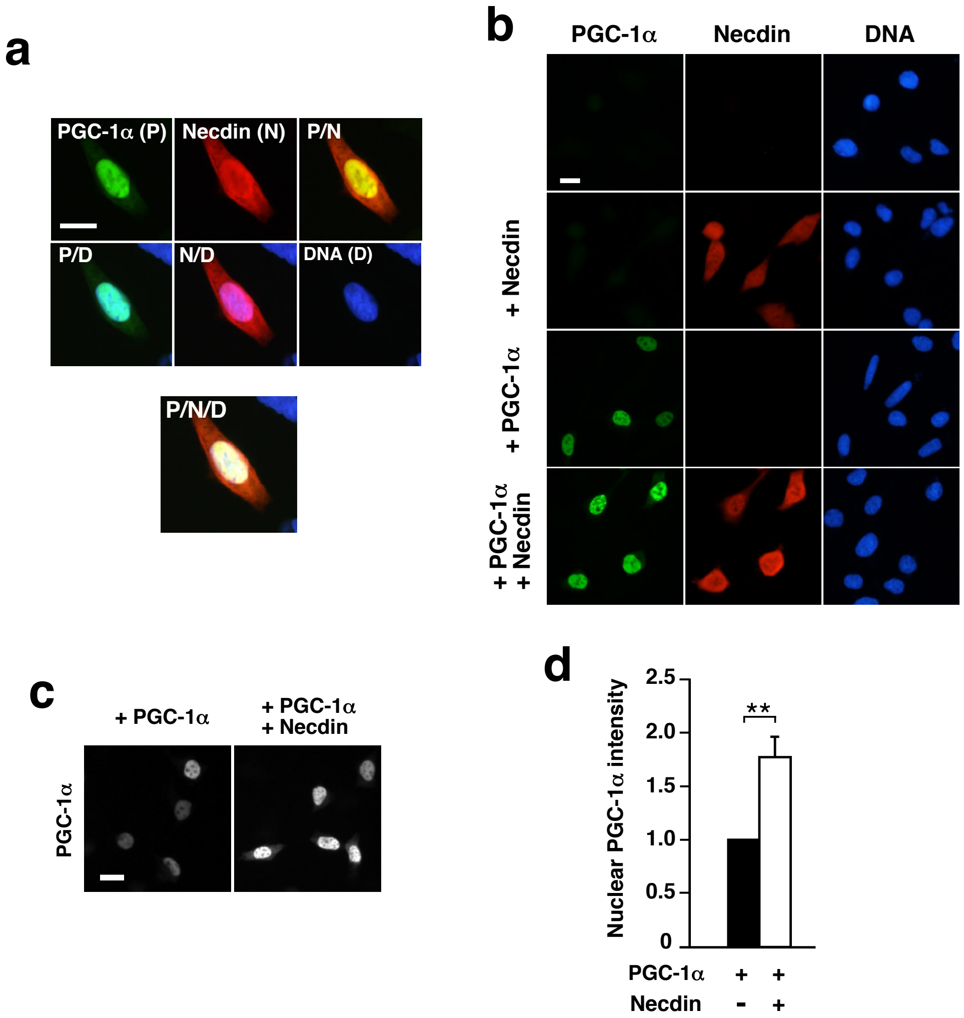

Supplementary Figure 3 | Necdin and PGC-1α are colocalized in the nucleus of transfected HEK293A cells. (**a**) HEK293A cells were transfected with PGC-1α and necdin cDNAs, and double immunostained for PGC-1α and necdin. Nuclear DNA (DNA) was counterstained with Hoechst 33342. Images were merged for their nuclear colocalization. (**b-d**) HEK293A transfected with cDNA for necdin, PGC-1α, or both was immunostained for necdin and PGC-1α (**b**), and nuclear PGC-1α signal intensities were measured by fluorescence microphotometry (**c**) and quantified (**d**)($n = 3$; mean ± s.e.m.; $**P < 0.01$; Student's t test). Scale bars, 20 μm.

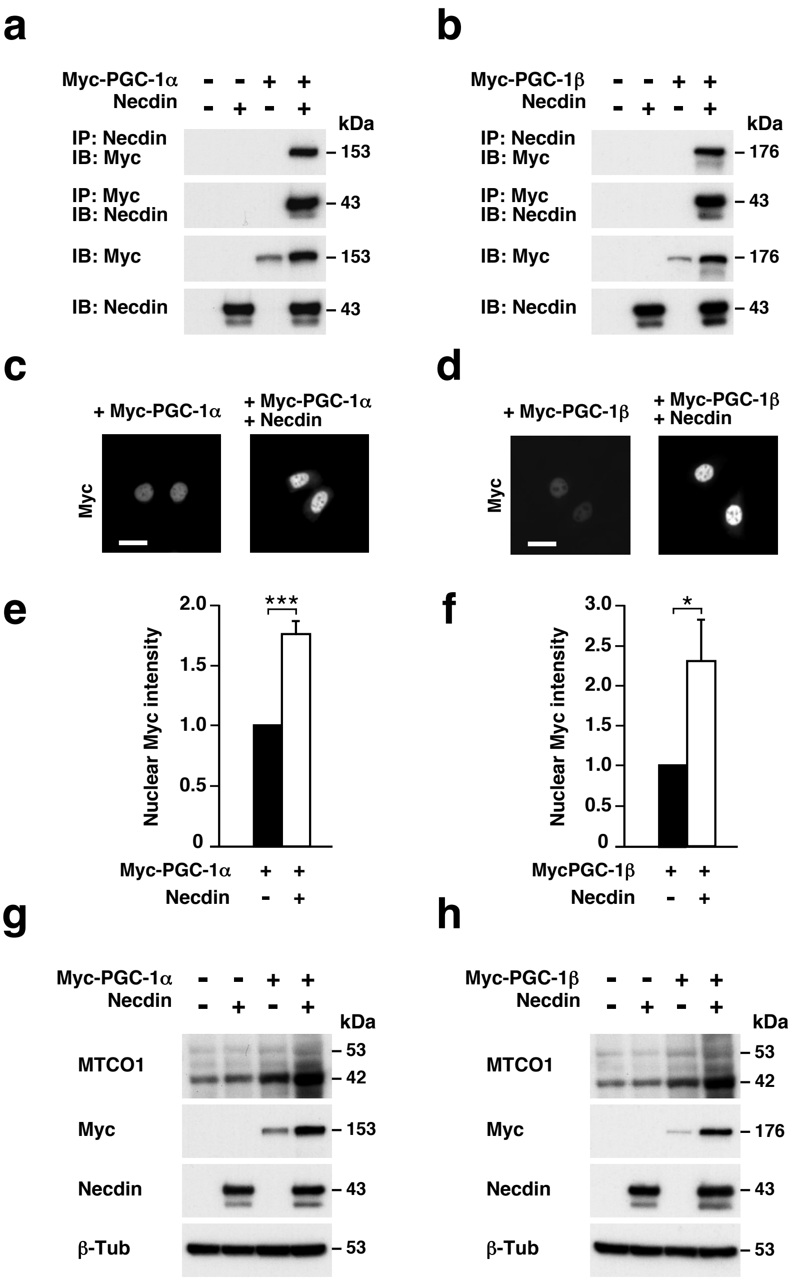

Supplementary Figure 4 | Necdin stabilizes PGC-1β and promotes PGC-1β-mediated MTCO1 expression. (**a**, **b**) HEK293A cells transfected with combinations of cDNAs for necdin, Myc-tagged PGC-1α (**a**), and Myc-tagged PGC-1β (**b**) were incubated for 24 h, and expressed proteins were immunoprecipitated (IP) and immunoblotted (IB) with antibodies to necdin and Myc. (**c**-**f**) Nuclear Myc signal intensities in HEK293A transfected with cDNAs for necdin and Myc-tagged PGC-1α (**c**) or Myc-tagged PGC-1β (**d**) were measured by fluorescence microphotometry and quantified (**e, f**) (n = 4; mean ± s.e.m.; *P < 0.05, ***P < 0.005; Student's t test). Scale bars, 20 μm. (**g**, **h**) HEK293A cells transfected with combinations of cDNAs for necdin, Myc-tagged PGC-1α (**g**), and Myc-tagged PGC-1β (**h**) were harvested 72 h post-transfection and analyzed by Western blotting for expression of MTCO1, Myc, necdin, and loading control β-tubulin (β-Tub).

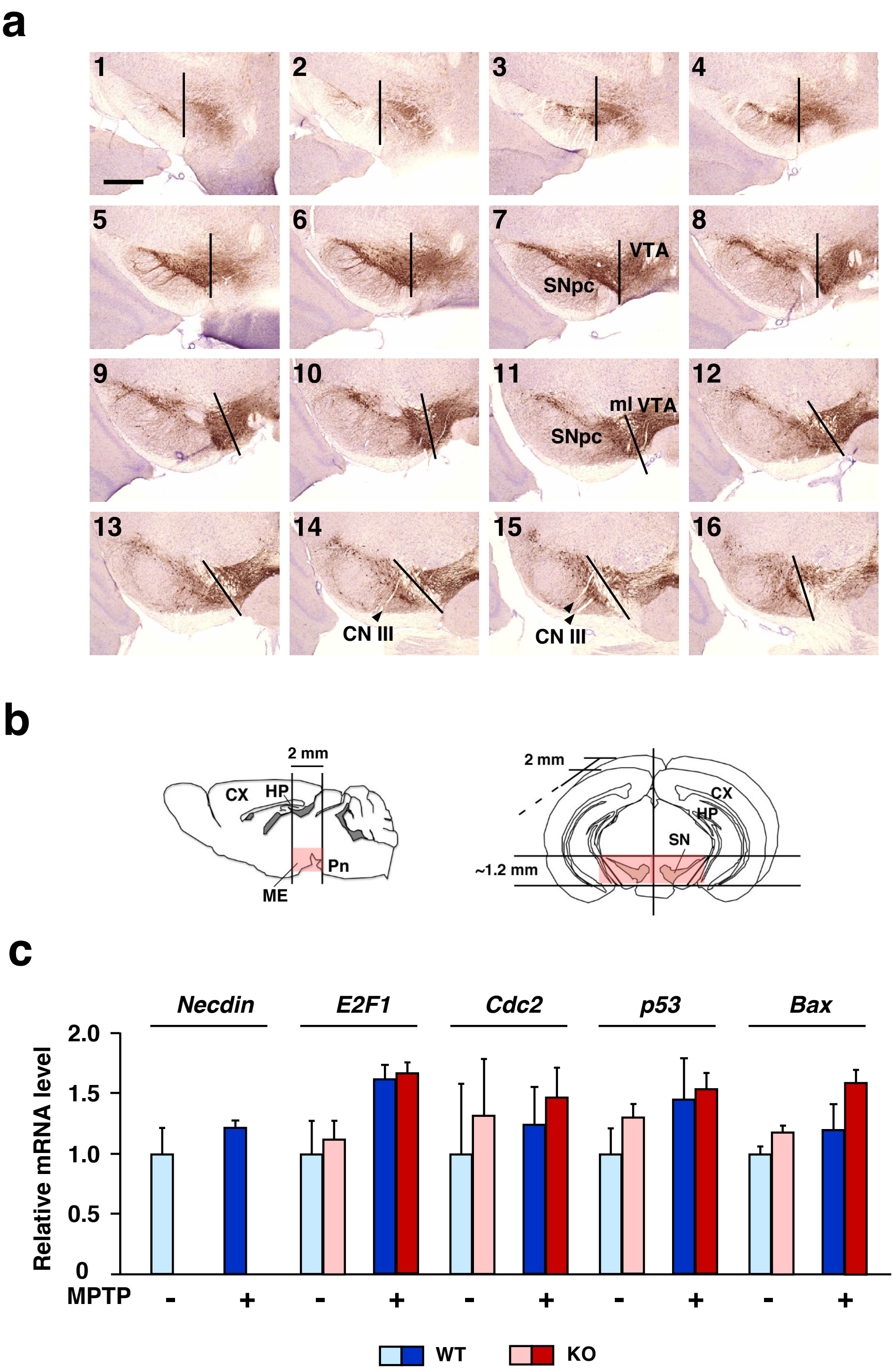

Supplementary Figure 5 | Immunohistochemical and Western blot analyses of the SN regions. (**a**) Brain sections were immunostained for TH by DAB staining followed by Nissl counterstaining. TH^{+} cells in the SNpc (black lines indicate the medial borders) were counted. The ventral tegmental area (VTA), medial lemniscus (ml) and oculomotor nerve (CN III) were used as makers for the SN extent. Scale bar, 500 μm. (**b**) Tissue dissection for Western blot analysis. Brain slices (2 mm thick) were prepared, and ventral midbrain areas (~1.2 mm wide) containing SN were collected. CX, cortex; HP, hippocampus; ME, median eminence; Pn, pontine nucleus; SN, substantia nigra. (**c**) mRNA expression levels of necdin-associated proapoptotic genes in the SN of wild-type (WT) and necdin-null mice (KO)(C57BL/6J strain) treated with or without MPTP were analyzed (*n* = 3-4).

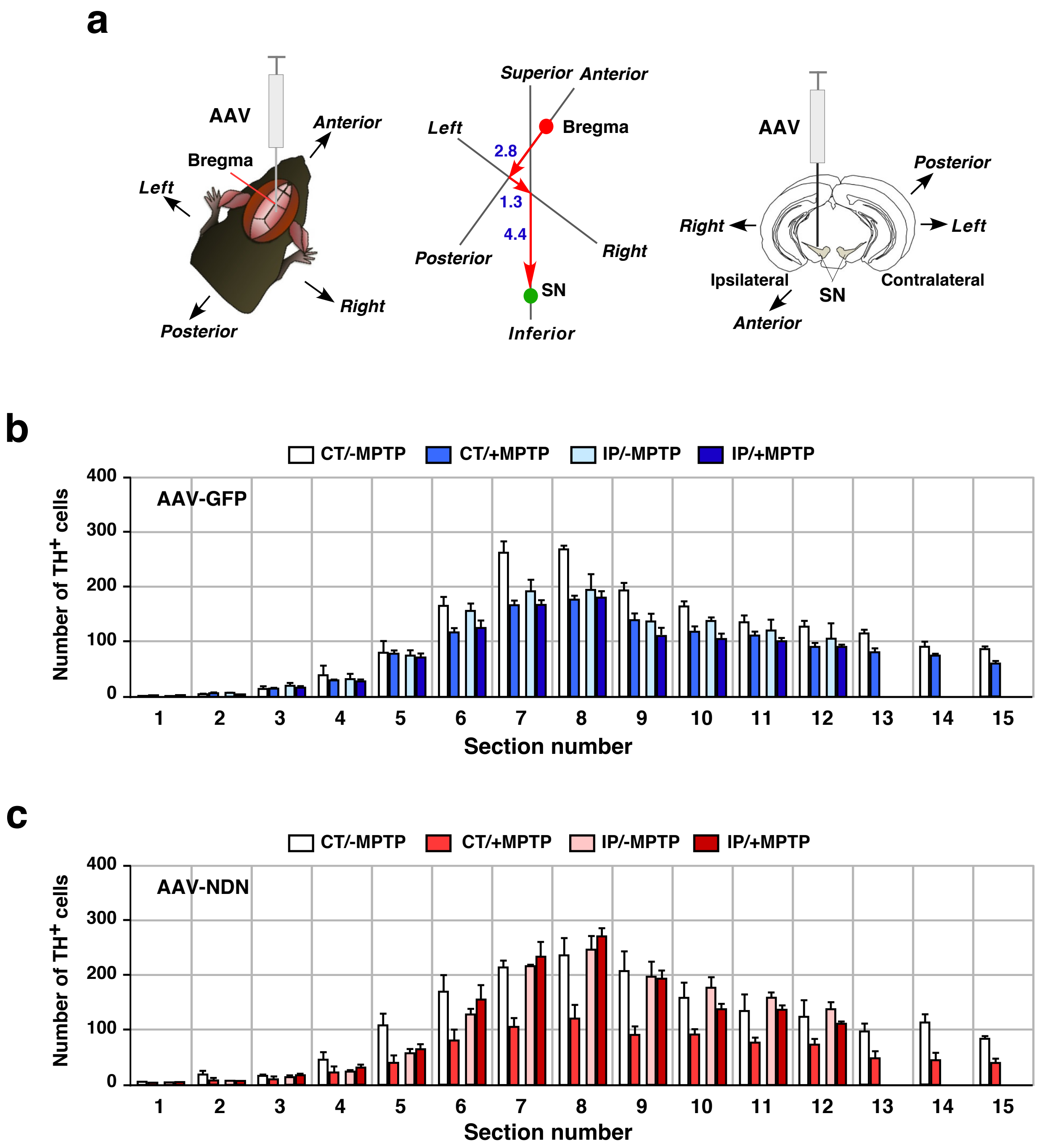

Supplementary Figure 6 | AAV-mediated *necdin* gene transfer into SN neurons and quantification of TH-expressing dopaminergic neurons in MPTP-induced PD mouse model. (**a**) Diagrams of AAV injection. AAV viruses (2 μl suspension) were stereotaxically injected into the SN of adult male C57BL/6J mice. Numbers (in mm), stereotaxic coordinates relative to the bregma. (**b, c**) TH^+ dopaminergic cells in the SNpc sections (12 sections, section number, 1-12) were counted in AAV-GFP (**b**) and AAV-NDN (**c**) mice without (-MPTP) or with (+MPTP) MPTP treatment. The extent of AAV-infected areas in the ipsilateral SNpc (section number 1-12) was judged by immunohistochemistry for GFP or necdin.

Supplementary Figure 7

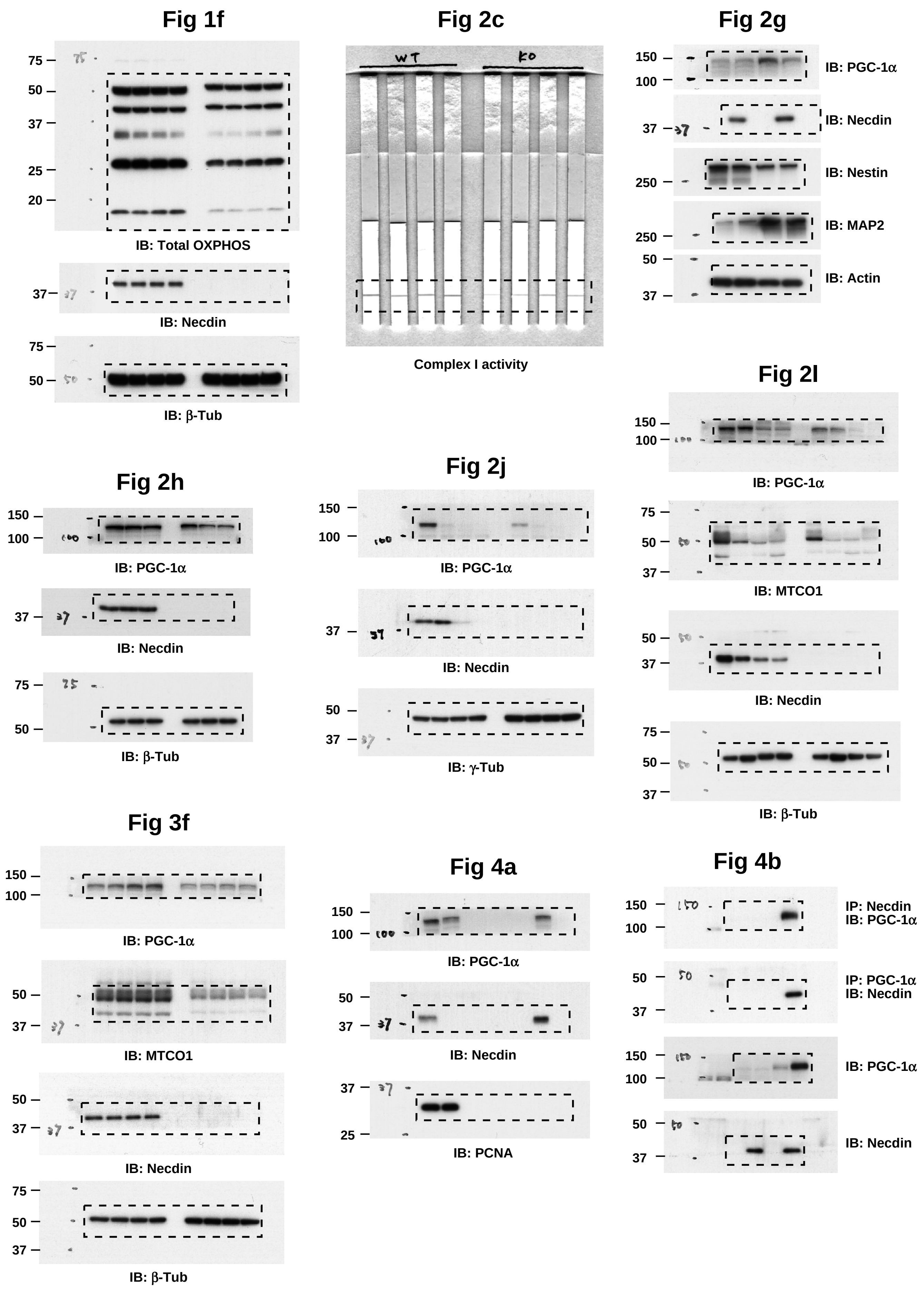

Supplementary Figure 7 (continued)

Fig 4d

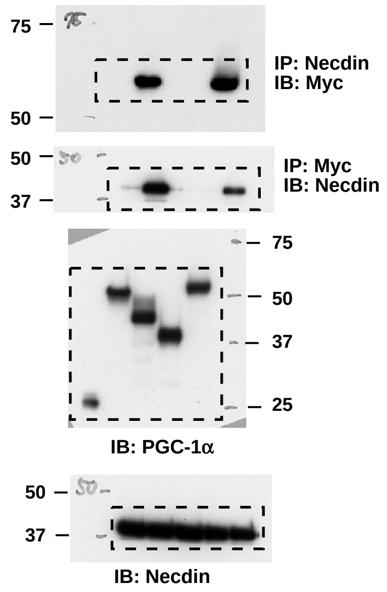

Fig 4e

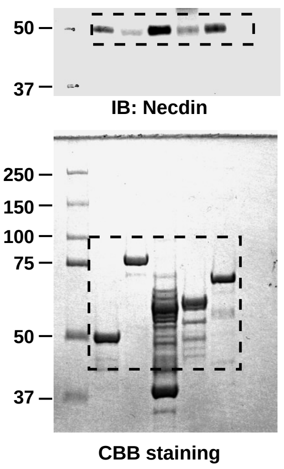

Fig 4f

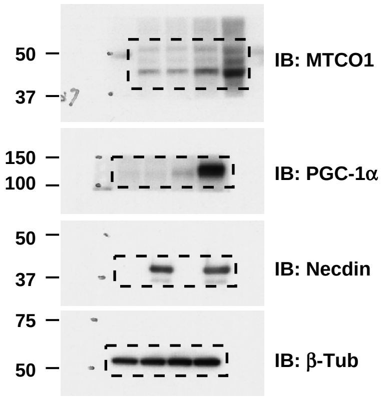

Fig 4i

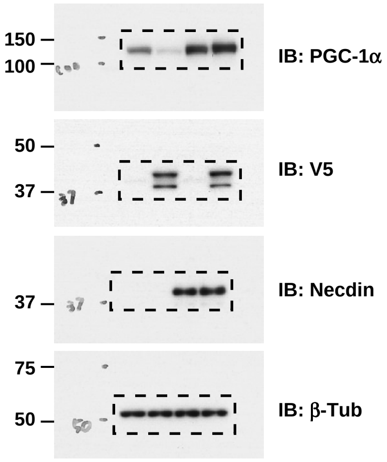

Fig 4h

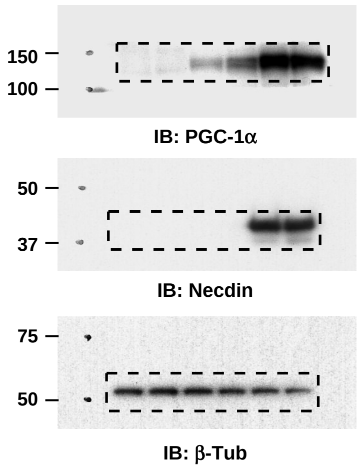

Fig 4g

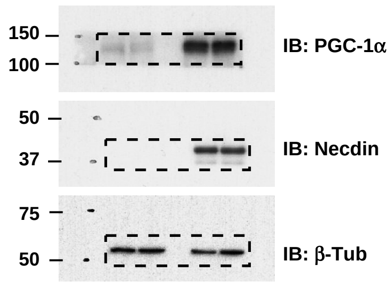

Fig 4j

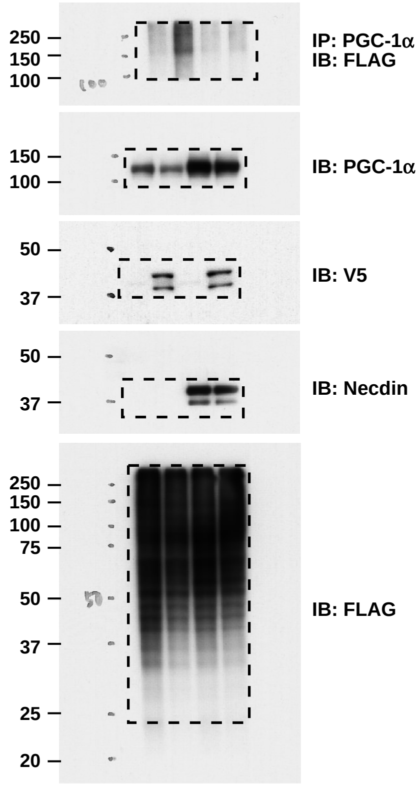

Fig 4k

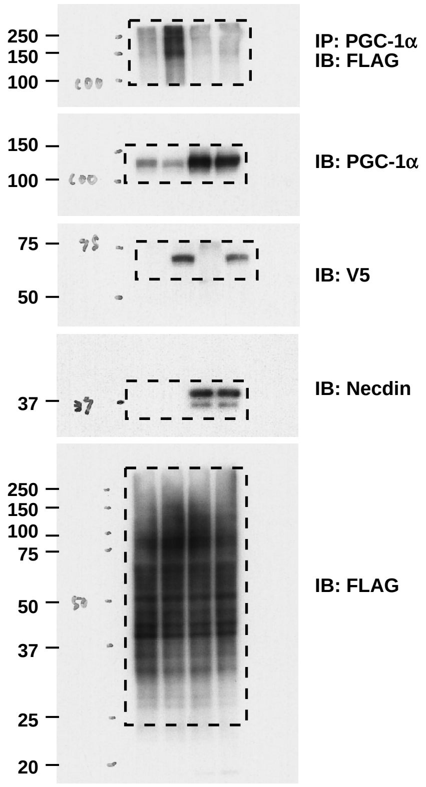

Fig 5a

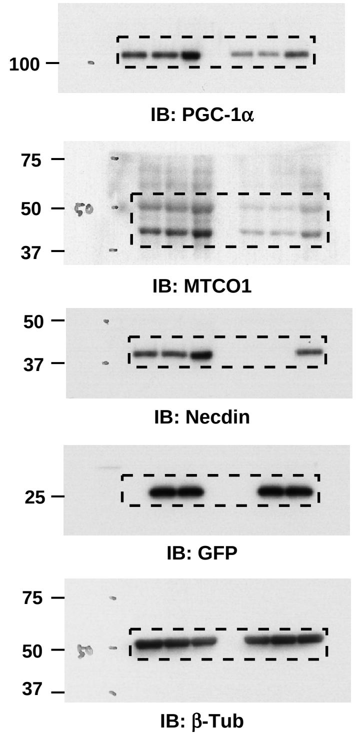

Supplementary Figure 7 (continued)

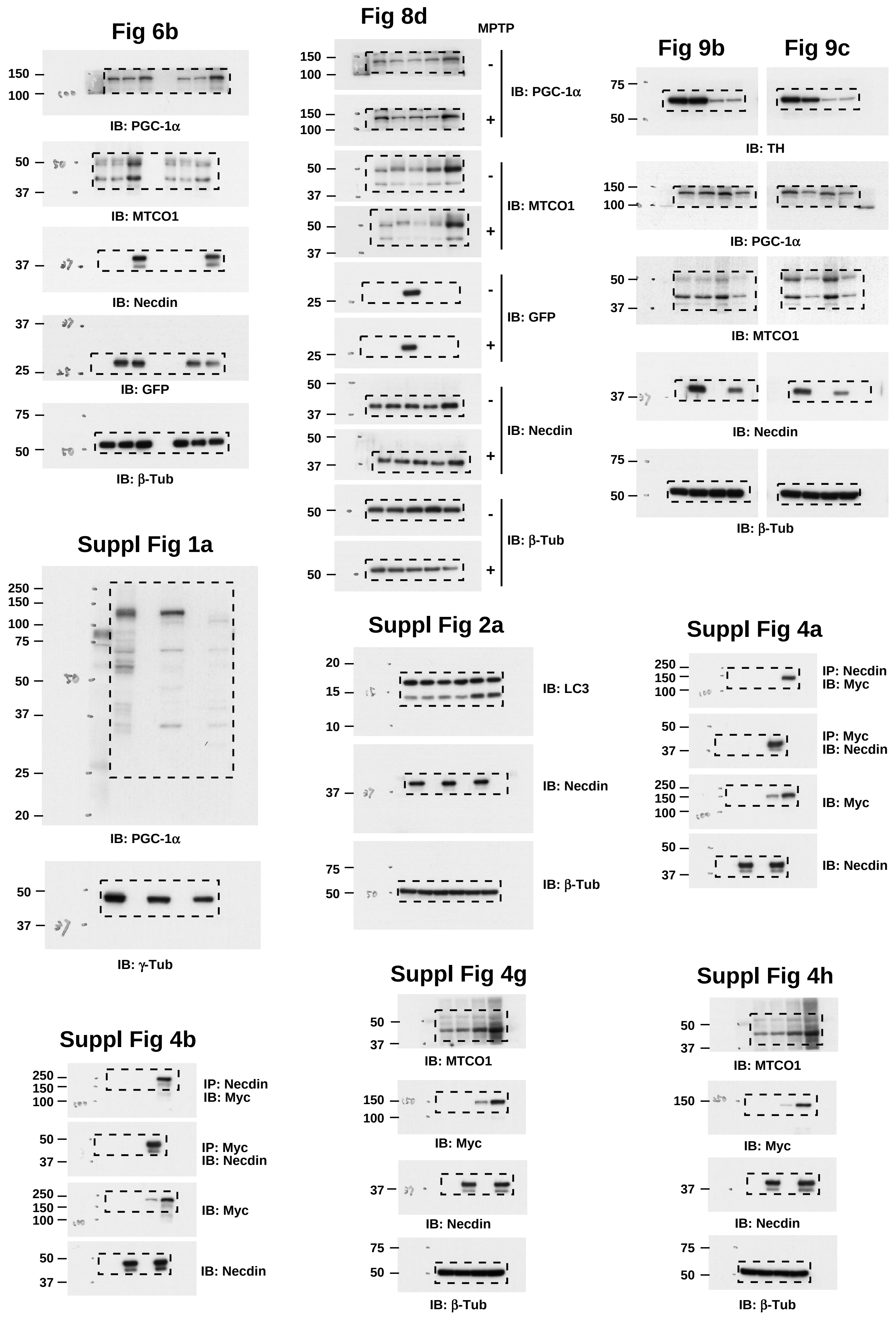

Supplementary Table 1 Microarray data on mitochondrion-related gene expression

Mitochondrion-related genes, KO < WT (*P* < 0.05)

Gene symbol	Per array normalized intensity (Test, KO)			Per array normalized intensity (Control, WT)			Average intensity				
	KO1	KO2	KO3	WT1	WT2	WT3	KO	WT	Ratio	log2ratio	*P*-value
**Timm9**	**44**	**48**	**39**	**61**	**53**	**62**	**43**	**59**	**0.74**	**-0.44**	**0.021**
**Cyc1**	**379**	**441**	**402**	**531**	**505**	**564**	**408**	**533**	**0.76**	**-0.39**	**0.008**
Mrpl45	60	69	61	82	75	80	63	79	0.80	-0.31	0.016
Ndufaf4	121	134	119	152	153	146	125	150	0.83	-0.27	0.009
Ttc19	25	32	23	38	36	34	27	36	0.74	-0.44	0.037
Mul1	37	37	33	44	42	41	36	43	0.84	-0.25	0.013
Mdh2	591	614	519	690	694	689	575	691	0.83	-0.27	0.021
Mrpl37	70	76	68	78	87	80	71	82	0.87	-0.20	0.047
Synj2bp	83	83	76	97	107	100	81	102	0.79	-0.33	0.004
Bcs1l	36	36	35	42	42	41	36	42	0.86	-0.23	0.001
Slc25a1	167	177	156	222	200	182	167	202	0.83	-0.27	0.047
Mrpl1	80	73	78	96	87	95	77	93	0.83	-0.27	0.009
Sirt5	28	28	30	41	37	41	29	39	0.73	-0.45	0.001
Ndufv1	280	283	288	342	319	358	284	340	0.84	-0.26	0.007
Slc25a19	57	59	60	66	72	76	59	71	0.82	-0.28	0.010
Grpel2	84	81	83	87	95	97	83	93	0.89	-0.17	0.025
Mrpl28	125	157	157	194	195	205	146	198	0.74	-0.43	0.018
Ak2	71	88	88	110	114	116	82	113	0.73	-0.46	0.012
Mtx2	163	197	189	230	228	232	183	230	0.80	-0.33	0.016
Mpst	145	169	163	190	199	187	159	192	0.83	-0.27	0.019
Eci1	46	58	53	68	70	65	52	68	0.78	-0.36	0.019
**Ndufs3**	**322**	**426**	**389**	**506**	**483**	**467**	**379**	**485**	**0.78**	**-0.36**	**0.042**
Mrpl15	83	116	110	137	142	135	103	138	0.75	-0.42	0.049
Cox7a2l	352	453	384	539	520	531	396	530	0.75	-0.42	0.016
Ndufv2	857	1077	952	1250	1278	1262	962	1263	0.76	-0.39	0.014
Coq7	71	99	84	110	118	110	85	113	0.75	-0.41	0.040
**Atp5d**	**850**	**1069**	**938**	**1145**	**1238**	**1205**	**952**	**1196**	**0.80**	**-0.33**	**0.030**
Slc25a33	95	102	99	130	124	123	99	126	0.79	-0.35	0.001
**Tomm40**	**182**	**193**	**196**	**246**	**231**	**239**	**190**	**238**	**0.80**	**-0.32**	**0.001**
Oxa1l	196	212	206	240	252	237	205	243	0.84	-0.25	0.004
**Tomm20**	**430**	**453**	**441**	**514**	**535**	**535**	**442**	**528**	**0.84**	**-0.26**	**0.001**
Mrpl38	78	88	85	97	105	104	83	102	0.82	-0.29	0.010
Tfb1m	19	22	23	31	25	28	21	28	0.75	-0.41	0.028
Slc25a39	122	137	137	164	154	159	132	159	0.83	-0.27	0.012
Parl	129	156	148	188	173	167	144	176	0.82	-0.29	0.041
**Tomm22**	**258**	**311**	**298**	**403**	**380**	**357**	**289**	**380**	**0.76**	**-0.39**	**0.014**
Sfxn1	291	328	322	397	387	358	314	381	0.82	-0.28	0.017
Mtch2	344	393	424	452	484	468	387	468	0.83	-0.27	0.039
Mpv17	32	38	39	44	46	49	36	46	0.79	-0.35	0.024
Mrpl3	176	197	188	203	225	211	187	213	0.88	-0.19	0.044
Mrps23	164	216	213	253	297	253	197	268	0.74	-0.44	0.041
**Atp5c1**	**442**	**460**	**493**	**502**	**573**	**544**	**465**	**540**	**0.86**	**-0.22**	**0.041**
Slc25a38	193	211	212	229	253	247	205	243	0.84	-0.24	0.016
Gcdh	34	36	38	43	49	42	36	45	0.80	-0.31	0.016
Mterfd2	76	79	90	97	111	96	82	101	0.81	-0.31	0.038
Pmpcb	221	221	238	270	278	242	227	263	0.86	-0.22	0.037
Poldip2	132	118	133	151	150	146	128	149	0.86	-0.22	0.017
Ddx28	65	56	65	74	78	76	62	76	0.82	-0.29	0.018
Phb2	365	341	396	439	425	424	367	429	0.86	-0.23	0.024
Spns1	49	49	54	69	60	59	51	63	0.81	-0.31	0.021
Bbc3	45	45	49	52	51	50	47	51	0.91	-0.13	0.041
Suclg1	214	227	249	289	271	284	230	281	0.82	-0.29	0.013
Chchd3	229	236	269	298	308	299	244	301	0.81	-0.30	0.013
**Timm50**	**156**	**165**	**193**	**216**	**209**	**210**	**172**	**211**	**0.81**	**-0.30**	**0.030**
**Atp5f1**	**870**	**898**	**1061**	**1175**	**1141**	**1109**	**943**	**1142**	**0.83**	**-0.28**	**0.038**
Capn10	66	67	72	76	80	78	68	78	0.88	-0.19	0.012
Apool	34	38	45	48	54	51	39	51	0.76	-0.40	0.033
Mrps7	38	40	44	45	48	48	41	47	0.87	-0.21	0.038
Prdx1	620	597	672	718	769	775	630	754	0.84	-0.26	0.013
Psen2	16	17	19	20	22	23	17	22	0.80	-0.33	0.018
Bckdhb	101	101	114	118	123	132	105	124	0.85	-0.24	0.037

Mitochondrion-related genes, KO > WT (*P* < 0.05)

Gene symbol	Per array normalized intensity (Test, KO)			Per array normalized intensity (Control, WT)			Average intensity				
	KO1	KO2	KO3	WT1	WT2	WT3	KO	WT	Ratio	log2ratio	*P*-value
Nipsnap1	31	32	28	26	25	26	31	26	1.19	0.26	0.014
Slc25a23	336	336	293	261	250	262	322	258	1.25	0.32	0.010
Ccdc109a	30	30	28	27	26	24	29	26	1.15	0.20	0.028
Maoa	655	665	587	459	422	425	635	435	1.46	0.55	0.001
Esr1	165	168	159	156	147	149	164	151	1.09	0.12	0.029
Gyk	31	30	30	24	25	29	30	26	1.18	0.23	0.045
Cpt1c	908	817	839	723	737	797	854	752	1.14	0.18	0.043
Slc25a35	250	240	230	214	208	227	240	216	1.11	0.15	0.041
Sh3glb1	249	219	239	200	207	203	236	203	1.16	0.21	0.020
Etfa	134	122	125	112	110	114	127	112	1.13	0.18	0.013
Bnip3	224	239	262	160	188	189	242	179	1.35	0.43	0.014
Hk2	40	38	49	32	35	33	43	33	1.28	0.36	0.040
Mrpl13	163	158	165	139	141	138	162	139	1.16	0.22	0.000
Slc27a1	80	73	83	54	57	56	79	55	1.42	0.51	0.001
Glud1	335	324	338	283	298	293	332	292	1.14	0.19	0.002

Supplementary Table 2. Primers used for quantitative RT-PCR analysis

Gene (Accession #)		Primer sequence
Tomm20 (NM_024214)	Forward	GGATTTACAGGTCTGCTATG
	Reverse	CCAAGCTAAAACTTTCCAAC
Tomm22 (NM_172609)	Forward	CATGATCCTGGTTCTTCCCGTTGT
	Reverse	AGGTAGAGCCCCTGGCATTC
Tomm40 (NM_016871)	Forward	TGAACAGTAACTGGATCGTG
	Reverse	GGAGGACATCAAGTCTTTCC
Timm9 (NM_013896)	Forward	AATATGGCTGCACAGATACC
	Reverse	TTCAGGTTTCACCTCTCTTG
Timm50 (NM_025616)	Forward	GACATCTCCTGTCTGAATCGG
	Reverse	GTGGGTCATCCTCTAGAGCA
Ndufs3 (NM_026688)	Forward	GCTTCGAGGGACATCCTTTC
	Reverse	AGTTACTTGGTTTCAGGCTTCT
Cyt-c (NM_007808)	Forward	AACCCATGAAGTACATGTGG
	Reverse	TGTAACGGAAGACAGATGGT
Atp5c1 (NM_020615)	Forward	CATGGACAACGCCAGCAAGA
	Reverse	TTTACCTCTTGTCTGAGGATGCAAC
Atp5d (NM_025313)	Forward	GCTGAAGAAGCTGTGACACT
	Reverse	TTGGCCTCAATACGGATCTG
Atp5f1 (NM_009725)	Forward	GTCGCAAGGAGGAAGAACAC
	Reverse	TCACATAATTGGCTGAGCTTGA
Nrf1 (NM_010938)	Forward	AGGAACCCTCAGTCTCACGA
	Reverse	GTCCTCACAGAGTCTTCTGACAC
Gabpa (NM_008065)	Forward	GATGAGTTAGCCAACTGCATTC
	Reverse	CCTCCCTTCTACAAAAGACTCA
Tfam (NM_009360)	Forward	AGCTGGTGTTAGCATACGGA
	Reverse	GGTGGCAAATTCGGAAGAGG
Ppargc1a (NM_008904)	Forward	TATACTTTACGCAGGTCGAA
	Reverse	ACAGAGAGTGTAAAGTAGGAG
Ndn (NM_010882)	Forward	AGGACCTGAGCGACCCTAAC
	Reverse	TGCTGCAGGATTTTAGGGTCAAC
E2F1 (NM_001291105)	Forward	ATGGAAGAGGACCAACTGTC
	Reverse	CCTGAATCCCTAGGCTTCTG
Cdc2 (NM_007659)	Forward	GTCAAGAACCTGGACGAGAA
	Reverse	GAGCCAACGGTAAACAACAC
p53 (NM_001127233)	Forward	CACTGGAGTCTTCAAGTGTG
	Reverse	GTCTGGGACAGCCAAGTCTG
Bax (NM_007527)	Forward	CTGAGCTGACCTTGGAGC
	Reverse	GACTCCAGCCACAAAGATG
Gapdh (NM_001289726)	Forward	ACAGAGAGTGTAAAGTAGGAG
	Reverse	GCAGCGAACTTTATTGATGGTA
